# Macrophage global metabolomics identifies cholestenone as host/pathogen cometabolite present in human *Mycobacterium tuberculosis* infection

**DOI:** 10.1172/JCI152509

**Published:** 2022-02-01

**Authors:** Pallavi Chandra, Héloise Coullon, Mansi Agarwal, Charles W. Goss, Jennifer A. Philips

**Affiliations:** 1 Division of Infectious Diseases, Department of Medicine,; 2 Department of Molecular Microbiology, and; 3 Division of Biostatistics, Washington University School of Medicine, St. Louis, Missouri, USA.

**Keywords:** Infectious disease, Microbiology, Cholesterol, Macrophages, Tuberculosis

## Abstract

*Mycobacterium tuberculosis* (*M. tuberculosis*) causes an enormous burden of disease worldwide. As a central aspect of its pathogenesis, *M. tuberculosis* grows in macrophages, and host and microbe influence each other’s metabolism. To define the metabolic impact of *M. tuberculosis* infection, we performed global metabolic profiling of *M. tuberculosis*–infected macrophages. *M. tuberculosis* induced metabolic hallmarks of inflammatory macrophages and a prominent signature of cholesterol metabolism. We found that infected macrophages accumulate cholestenone, a mycobacterial-derived, oxidized derivative of cholesterol. We demonstrated that the accumulation of cholestenone in infected macrophages depended on the *M. tuberculosis* enzyme 3**β**-hydroxysteroid dehydrogenase (3**β**-Hsd) and correlated with pathogen burden. Because cholestenone is not a substantial human metabolite, we hypothesized it might be diagnostic of *M. tuberculosis* infection in clinical samples. Indeed, in 2 geographically distinct cohorts, sputum cholestenone levels distinguished subjects with tuberculosis (TB) from TB-negative controls who presented with TB-like symptoms. We also found country-specific detection of cholestenone in plasma samples from *M. tuberculosis*–infected subjects. While cholestenone was previously thought to be an intermediate required for cholesterol degradation by *M. tuberculosis*, we found that *M. tuberculosis* can utilize cholesterol for growth without making cholestenone. Thus, the accumulation of cholestenone in clinical samples suggests it has an alternative role in pathogenesis and could be a clinically useful biomarker of TB infection.

## Introduction

*Mycobacterium tuberculosis (M. tuberculosis)*, the causative agent of tuberculosis (TB), is an intracellular pathogen that causes an enormous worldwide burden of disease. It is estimated that 1.7 billion people are latently infected with *M. tuberculosis*, and in 2017 there were an estimated 10 million new cases of TB and 1.4 million deaths (1). The diagnosis of active TB is often challenging, leading to long delays in treatment. Treatment generally requires multiple antibiotics for a minimum of 6 months, and relapse occurs in 3% to 5% of treated patients (2). In some patients, long courses of therapy expose patients unnecessarily to side effects of antibiotics, while in other patients, stopping therapy prematurely leads to relapse. As such, there is a large unmet medical need for new therapeutics; rapid, sensitive, and affordable diagnostic methods; and biomarkers to allow tailored therapy and to guide clinical decision making.

As a central aspect of its pathogenesis, *M. tuberculosis* grows in macrophages, and host and microbe influence each other’s metabolism. Previous work in our lab investigated immuno-metabolic dynamics of the host during *M. tuberculosis* infection. We showed that *M. tuberculosis* induces miR-33 to control host pathways critical for its intracellular survival, including lipid metabolism and autophagy (3). We also showed that intracellular growth of *M. tuberculosis* depends on macrophage fatty acid catabolism and that inhibiting host fatty acid catabolism enhances immune effector functions against *M. tuberculosis* (4). Given the increasing appreciation of the link between metabolism and immunity, we performed unbiased metabolomics to explore perturbations in metabolic pathways during *M. tuberculosis* infection. We found that *M. tuberculosis*–infected macrophages preferentially utilize glucose, channel TCA cycle intermediates for itaconate production, and induce perturbations in redox homeostasis. As previous reports have shown, such metabolic changes support host antimicrobial functions by allowing rapid ATP and NADPH production, enhancing oxidative stress and inflammation, and providing raw materials for plasma membrane synthesis and protein export (5–7). In addition to these immuno-metabolic changes, we found a prominent metabolic signature of cholesterol metabolism, with cholestenone (4-cholesten-3-one) the second most discriminating metabolite between infected and uninfected cells.

Host cholesterol can serve as a carbon source that feeds mycobacterial central metabolism and the biosynthesis of methyl-branched fatty acids (8, 9). *M. tuberculosis* induces the formation of lipid-droplet–filled or foamy macrophages (10–13). In these foamy macrophages, *M. tuberculosis*–containing phagosomes are in close approximation to host lipid droplets (11). *M. tuberculosis* also grows extracellularly in the lipid-rich caseum of necrotic granulomas (14). Cholesterol utilization by *M. tuberculosis* has been linked to dormancy and persistence (8, 15). *M. tuberculosis* oxidizes host cholesterol to cholestenone, which is thought to be a necessary intermediate in cholesterol degradation. *M. tuberculosis* is able to completely degrade cholesterol through a process involving enzymes of the KstR1 regulon, which degrade the side chain and A/B ring, and the KstR2 regulon, which metabolize the C/D ring (16). Humans do not have a similar pathway to degrade cholesterol. Instead, cholesterol serves as a critical component of cellular membranes and is used for biosynthesis of bile acids, steroid hormones, and vitamin D. This raises the possibility that *M. tuberculosis*–specific cholesterol metabolites might be involved in pathogenesis and serve as unique biomarkers of infection. This led us to investigate the mechanism of cholesterol oxidation and the potential utility of cholesterol metabolites as TB biomarkers. Indeed, using clinical samples from 2 cohorts of patients, recruited from Peru and Vietnam, we found that cholestenone is a candidate biomarker of TB infection.

## Results

### Global metabolomics reveals immuno-metabolic changes and a signature of cholesterol metabolism associated with M. tuberculosis infection.

**Figure 1:**
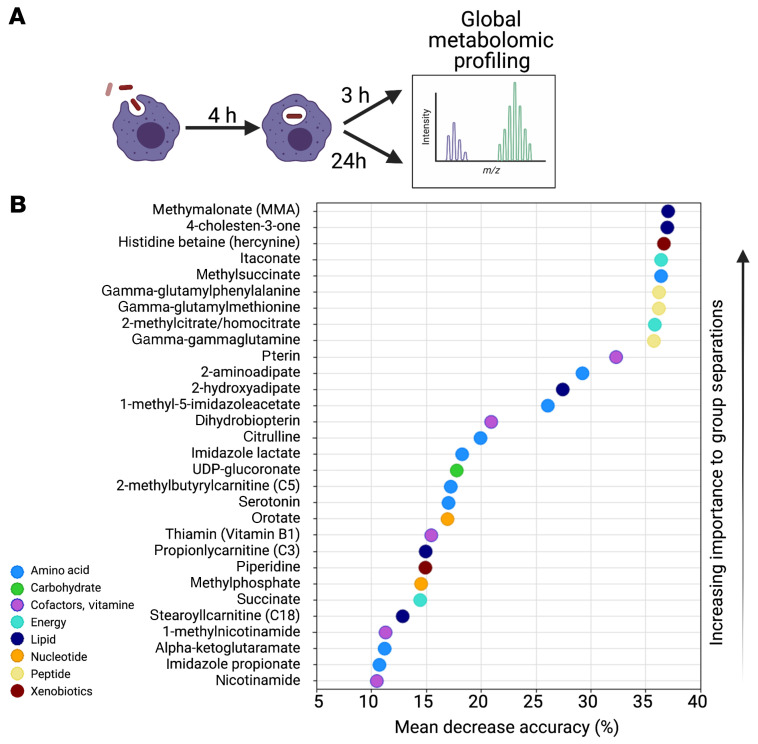
Global metabolomic profiling identifies metabolites that discriminate *M.* *tuberculosis*–infected from uninfected macrophages. (**A**) Murine BMDMs were incubated with *M. tuberculosis* H37Rv at MOI 5 for 4 hours after which extracellular bacteria were removed. Infected macrophages were harvested 3 hpi and 24 hpi for metabolomics analyses. At each time point, uninfected cells were used as controls. (**B**) A biochemical importance plot indicating the top 30 metabolites that differentiate between uninfected and *M. tuberculosis*–infected macrophages. The mean decrease accuracy quantifies the importance of a metabolite to the prediction accuracy of the model. A higher value shows that the metabolite has more importance to group separation. The color of each molecule indicates the metabolic pathway with which it is associated. This plot is derived from random forest analysis of the metabolomics profile of 507 biochemicals obtained from 19 samples. See methods for additional details.

**Figure 2:**
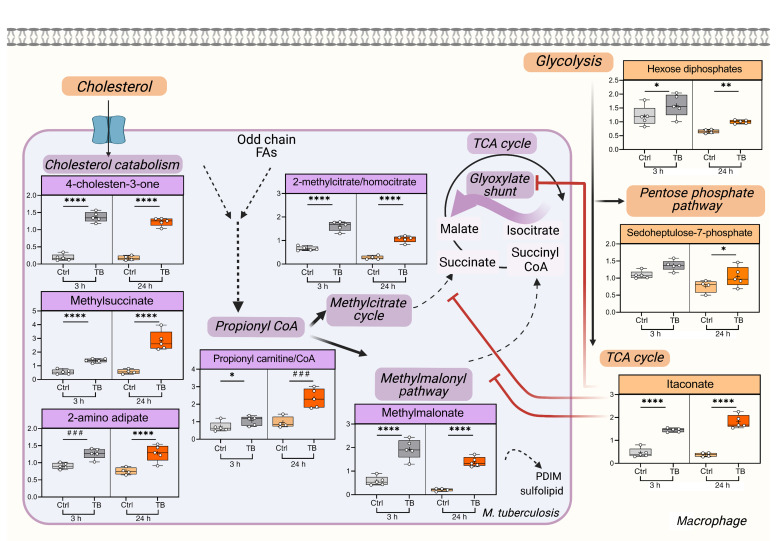
Metabolic signature of *M. tuberculosis* infection reflects changes in cholesterol metabolism. Schematic summarizing the relationship between key metabolic pathways and biochemicals that changed in response to infection. Metabolites that are likely from macrophages are orange, and those from *M. tuberculosis* are purple. Box plots show scaled intensity for relevant metabolites from the metabolomics screen in uninfected (Ctrl) and *M. tuberculosis*–infected (TB) samples 3 hpi and 24 hpi. Box plots show median as a line and mean as ‘+’, and each dot represents data from a single replicate. Statistical significance was calculated using ANOVA for all analytes identified in the screen. *P < 0.05, **P < 0.01, ^###^*P* ≤ 0.003, and ****P < 0.0001. Also see Supplemental Table 1 and Supplemental Text.

To identify a metabolic signature of *M. tuberculosis* infection, we globally profiled metabolites from *M. tuberculosis*–infected bone marrow–derived macrophages (BMDMs) at 7 and 28 hours postinfection (hpi) compared with uninfected cells (Figure 1A). Over 500 metabolites were reported from pathways involved in carbohydrate, lipid, nucleotide, and amino acid metabolism, redox homeostasis, inflammation, and xenobiotics (Supplemental Table 1; supplemental material available online with this article; https://doi.org/10.1172/JCI152509DS1). Since metabolite extraction was performed without separating intracellular *M. tuberculosis* from the macrophages, the identified metabolites could be from host or pathogen. Principle component analysis demonstrated clear separation of infected and uninfected samples, indicative of distinct metabolic phenotypes (Supplemental Figure 1). Using random forest analysis, we identified 30 metabolites that distinguished *M. tuberculosis*–infected from uninfected macrophages and ranked them according to their predictive power (Figure 1B). While differences in any individual metabolite from this global screen would require further validation, the overall changes were consistent with the idea that *M. tuberculosis* infection induces a Warburg-like glycolytic shift in macrophage metabolism (17), enhances flux to the pentose phosphate pathway for NADPH production, elevates itaconate levels, and alters redox homeostasis (gamma-glutamyl amino acids and pterin derivatives) (Figure 1B, Figure 2, Supplemental Table 1, and Supplemental Figures 2–4, see Supplemental Material for details). Compared with controls, *M. tuberculosis*–infected macrophages showed elevated itaconate, the fourth most discriminating metabolite (Figure 1B and Figure 2). Classically activated macrophages have a characteristic TCA cycle breakpoint, which allows conversion of *cis*-aconitate to the antimicrobial metabolite itaconate by the enzyme Irg1 (7, 18). Irg1 protects against severe pathology in *M. tuberculosis*–infected mice (19). *M. tuberculosis* infection also enhanced levels of nucleotide sugars (UDP-glucuronate), which may be used by the host for glycosylation reactions or by *M. tuberculosis* to support cell wall biosynthesis (Supplemental Figure 2, Supplemental Text). Overall, infection-induced molecules suggested upregulation of pathways that are metabolic hallmarks of an inflammatory macrophage phenotype.

**Figure 3:**
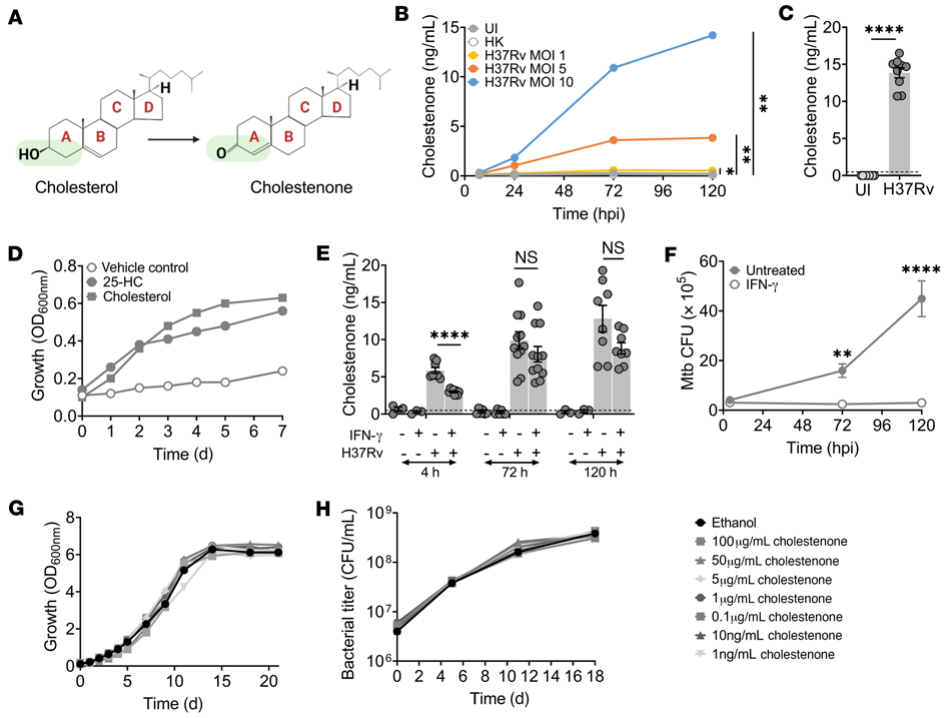
Macrophage cholestenone levels correlate with *M. tuberculosis* burden and duration of infection. (**A**) Chemical structures of cholesterol and cholestenone showing **A**–**D** rings (red text). The 3-hydroxyl group of cholesterol is dehydrogenated to a keto moiety in the A ring in cholestenone (highlighted in green). (**B**) Cholestenone was quantified from BMDMs infected with *M. tuberculosis* at MOI 1, 5, and 10, at 3, 24, 72, and 120 hpi. Uninfected (UI) cells or those infected with heat-killed *M. tuberculosis* (HK) were used as controls. Plot shows average of 2 independent experiments.*P = 0.03, **P = 0.007 for 120 hours, calculated using Mann-Whitney test. (**C**) Cholestenone levels in PMA-differentiated THP-1 macrophages that were uninfected or *M. tuberculosis*–infected at MOI 10, 72 hpi. (**D**) Growth of *M. tuberculosis* was compared in minimal medium supplemented with either a vehicle control, 100 μg/mL 25-HC, or 100 μg/mL cholesterol. Plot shows values from 1 experiment representative of 3 independent experiments. (**E**) Cholestenone abundance and (**F**) corresponding *M. tuberculosis* CFU in IFN-γ–activated and naive BMDMs that were uninfected or *M. tuberculosis*–infected at MOI 10 at the indicated time points. (**G** and **H**) The direct effect of cholestenone on *M. tuberculosis* growth was assessed in culture medium using absorbance measurements (**G**) and CFU (**H**). (**C**, **E**, and **F**) Plots show mean ± SEM from at least 3 independent experiments. **P = 0.007, ****P < 0.0001 calculated using Student’s *t* test (**C**), and 1-way ANOVA with Tukey’s multiple comparisons test (**E** and **F**). (**G** and **H**) Plots show average of 2 independent experiments. Also see Supplemental Figure 6. (**C** and **E**) The dotted line on the *y* axis represents the limit of detection accuracy as determined by the standard curve. For all macrophage experiments, one million cells were infected and the samples were extracted in 500 μL 80 % methanol solution containing 0.1 μg internal standard. The methods used for calculating cholestenone concentrations are detailed in the Supplemental Material.

The other notable signature that we observed was related to cholesterol utilization. The second most infection-discriminating metabolite was cholestenone, an oxidized derivative of cholesterol (Figure 2 and Figure 3A). Many of the other top metabolites, including methylsuccinate, methylcitrate, and 2-aminoadipate, were all previously enriched when *M. tuberculosis* was grown in vitro with cholesterol as compared with glycerol (ref. 20 and Figure 2). The top fifth discriminating metabolite was methylsuccinate, which may be generated by cholesterol C and D ring degradation (Figure 1B, Figure 2, and refs. 20–22). It can also be produced through catabolism of branched chain amino acids (23), but since we did not observe major changes in valine and isoleucine catabolites, the most likely source of methylsuccinate was degradation of host cholesterol by intracellular *M. tuberculosis* (Supplemental Table 1). Degradation of cholesterol and odd chain fatty acids by *M. tuberculosis* generates propionyl-CoA, which is in equilibrium with propionylcarnitine, another infection-induced metabolite identified in our screen (Figure 2). Propionyl-CoA feeds the methylmalonate pathway and methylcitrate cycle, which generate important products for synthesis of bacterial virulence lipids and central carbon metabolism (Figure 2). In response to infection, we detected significant increases of methylmalonate and 2-methylcitrate, 2 intermediates of these metabolic pathways. Interestingly, itaconate inhibits host and *M. tuberculosis* methylmalonyl-CoA mutase, which isomerizes methylmalonyl-CoA to succinyl-CoA (24). Itaconate also inhibits *M. tuberculosis* isocitrate lyase(s), which functions in the methylcitrate cycle (18, 25). Thus, elevated itaconate may contribute to the observed elevation in methylmalonate and methylcitrate. Overall, our metabolomics screen suggested dynamic immuno-metabolic changes attributable to host-pathogen interactions. The results also pointed to the oxidation and degradation of cholesterol by the bacilli. This is consistent with a previous study in *M. tuberculosis*–infected THP-1 macrophages in which the cholesterol metabolite 4,5-9,10-diseco-3-hydroxy-5,9,17-tri-oxoandrosta-1(10),2-diene-4-oic acid (DSHA) was detected (26). Our screening platform did not look for secosteroid and androstenedione-related metabolites that are intermediates in *M. tuberculosis* cholesterol degradation, such as DSHA, 4-androstenedione (ADD), 9-hydroxy-androsta-1,4-diene-3,17-dione (9OHADD), and 3-hydroxy-9,10-seconandrost-1,3,5(10)-triene-9,17-dione (3-HSA) (21). Despite this limitation, among a vast pool of host metabolites, the apparent signature of cholesterol metabolism was noteworthy. Since cholestenone is a unique metabolite that is likely to depend upon host and *M. tuberculosis* cometabolism, we further investigated its production during intracellular infection.

### Cholestenone abundance correlates with bacterial burden and duration of M. tuberculosis infection.

We infected BMDMs with *M. tuberculosis* at different multiplicities of infection (MOI) and estimated cholestenone abundance at various time points over 5 days of infection using mass spectrometry. During metabolite extraction, we spiked cell lysates with premeasured d_5_-cholestenone as an internal standard to verify the identity of the metabolite and to quantify its relative abundance (Supplemental Figure 5). We found that cholestenone levels increased with increasing bacterial burden and duration of infection (Figure 3B and Supplemental Figure 6A). As a control, we used heat-killed *M. tuberculosis*, which confirmed that cholestenone production requires live bacilli. We also verified that *M. tuberculosis* infection generated cholestenone in human cells by infecting THP-1 macrophages, a human monocytic cell line (Figure 3C). In addition, we examined the levels of methylmalonate, methylcitrate, and methylsuccinate, additional top differential metabolites related to cholesterol degradation, and itaconate. We included the corresponding internal standards for each metabolite during extraction (Supplemental Figure 5). We confirmed that *M. tuberculosis* infection enhanced the level of methylsuccinate and itaconate 72 hpi (Supplemental Figure 6B). We did not detect differences in the abundance of methylcitrate and methylmalonate 72 hpi, so their apparent elevation at earlier time points in the primary screen requires further validation. Thus, we confirmed a sustained increase in abundance of methylsuccinate, cholestenone, and itaconate in response to *M. tuberculosis* infection of BMDMs.

Recent reports show that IFN-γ induces the expression of cholesterol 25-hydroxylase, which converts cholesterol to the immunomodulatory oxysterol 25-hydroxycholesterol (25-HC) which can have antimicrobial activity (27, 28). Since IFN-γ activation also enhances the ability of macrophages to control *M. tuberculosis*, we investigated the ability of *M. tuberculosis* to metabolize 25-HC. First, we examined whether *M. tuberculosis* can use 25-HC as a carbon source and found that *M. tuberculosis* grew in minimal media supplemented with 25-HC as the sole carbon source (Figure 3D). Next, we infected IFN-γ–activated and untreated BMDMs, and compared cholestenone abundance at various time points of infection (Figure 3E). At the same time, we generated a standard curve to determine the linear range of the assay and to establish the concentration of cholestenone in samples (Supplemental Figure 7A). In addition, we quantified total cholesterol, *M. tuberculosis* burden, and macrophage cell viability at each time point (Figure 3F and Supplemental Figure 6). *M. tuberculosis* infected both naive and IFN-γ–activated macrophages equally. However, macrophages that had been pretreated with IFN-γ had significantly reduced cholestenone abundance early (4 hours) after infection. At 72 hpi and 120 hpi, cholestenone levels trended lower but were not significantly different between treated and untreated macrophages (Figure 3, E and F) even though bacterial CFUs were significantly lower in the IFN-γ–treated macrophages. Thus, IFN-γ treatment appears to influence the relationship between bacterial burden and cholestenone levels. Although total cholesterol levels were not affected by IFN-γ (Supplemental Figure 6), IFN-γ profoundly affects the intracellular niche of the *M. tuberculosis* as well as host metabolism, including altering the distribution of different host cholesterol pools (28). Thus, IFN-γ might alter cholestenone production or subsequent metabolism by affecting host or bacterial metabolism. To determine whether cholestenone could have direct antimicrobial activity against *M. tuberculosis*, we evaluated its effect on bacilli growing in broth culture. We found that cholestenone was not directly toxic to *M. tuberculosis*, even at concentrations in vast excess of what was found in macrophages (Figure 3, G and H). Taken together, our findings demonstrate that *M. tuberculosis* infection of IFN-γ–activated and naive macrophages leads to the production of cholestenone, an oxidized derivative of host cholesterol.

### 3β-hydroxysteroid dehydrogenase, not ChoD, converts cholesterol to cholestenone.

**Figure 4:**
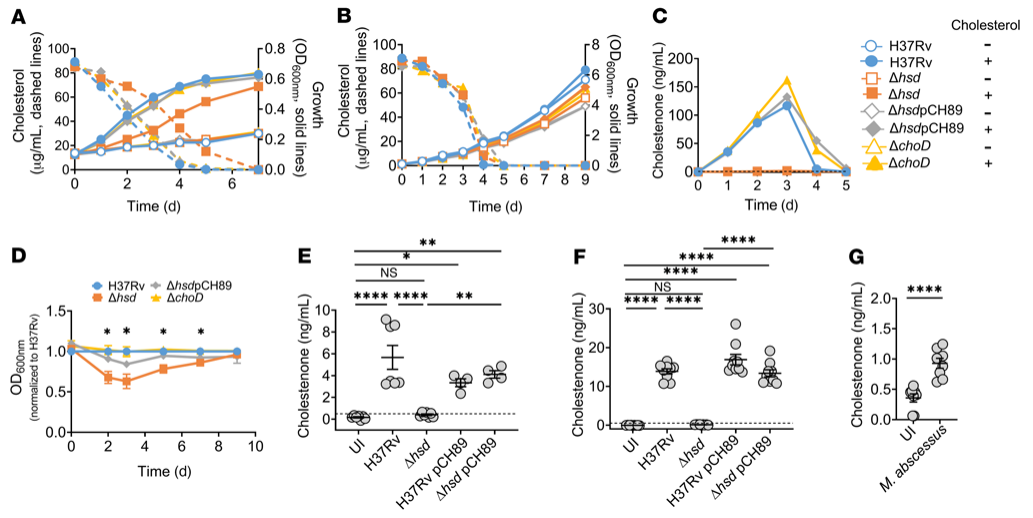
*M. tuberculosis* 3β-hydroxysteroid dehydrogenase, not ChoD, catalyzes oxidation of cholesterol to cholestenone. (**A** and **B**) Growth of H37Rv, Δ*hsd*, Δ*hsd* *att*:pCH89 (Δ*hsd*pCH89), and Δ*choD* in (**A**) minimal media and (**B**) 7H9 supplemented with OADC. Cultures supplemented with cholesterol are indicated by closed symbols, while open symbols represent cultures with the vehicle control. Cholesterol abundance in the cultures is represented with the dotted lines. (**C**) Cholestenone abundance measured during growth of H37Rv, Δ*hsd*, Δ*hsd*pCH89, and Δ*choD* in 7H9 supplemented with OADC and cholesterol (100 μg/mL). (**D**) Growth of H37Rv, Δ*hsd*, Δ*hsd*pCH89, and Δ*choD* in minimal media supplemented with cholesterol, expressed as the ratio of OD_600nm_ for each strain normalized to H37Rv. Plot shows the mean and SEM from 3 independent experiments, *P < 0.05 indicates significance between H37Rv and Δ*hsd* and was done using Student’s *t* test. (**E** and **F**) Cholestenone abundance at 72 hpi in (**E**) BMDMs and (**F**) PMA-differentiated THP-1 macrophages infected with WT *M. tuberculosis*, *Δhsd*, H37Rv *att*:pCH89 (H37RvpCH89), or Δ*hsd*pCH89. (**G**) Cholestenone abundance in BMDMs infected with *M*. *abscessus* at MOI 1, 24 hpi. (**A**–**C**) Graphs are representative of 3 independent experiments. (**E** and **G**) Data shows (**E**) mean ± SD from at least 2 independent experiments, and (**F** and **G**) SEM from 3 independent experiments. (**E**–**G**) *P = 0.01, **P ≤ 0.004, ****P < 0.0001 calculated using (**E** and **F**) 1-way ANOVA with Tukey’s multiple comparisons test and (**G**) Student’s *t* test. UI, uninfected. (**C**, **E**, and **F**) The dotted line on the *y* axis represents the limit of detection accuracy as determined by the standard curve. For all macrophage experiments, one million cells were infected and the samples were extracted in 500 μL 80 % methanol solution containing 0.1 μg internal standard. The methods used for calculating cholestenone concentrations are detailed in the Supplemental Material.

As far as we are aware, there is no mammalian enzyme that is reported to convert cholesterol to cholestenone. There is ambiguity in the literature as to the identity of the enzyme that is responsible for cholestenone production by *M. tuberculosis* and whether the oxidation step is required for cholesterol degradation. The enzymes that have been evaluated in previous studies are 3β-hydroxysteroid dehydrogenase (3β-Hsd, Rv1106c) and cholesterol oxidase (ChoD, Rv3409). Purified 3β-Hsd has cholesterol oxidase activity in vitro (29, 30). However, in the H37Rv strain background, a Δ*hsd* mutant metabolizes cholesterol to degradative intermediates and grows on cholesterol (31). ChoD (Rv3409) has been investigated as an alternative cholesterol oxidase, but the H37Rv Δ*hsd* Δ*choD* double mutant is also reported to grow on cholesterol (31). Given the apparent contradictory results, we sought to identify the cholesterol oxidase(s) in our strain and determine whether it was required for cholesterol degradation. We used oligonucleotide-mediated recombineering followed by Bxb1 integrase targeting (ORBIT) to delete *hsd* (Δ*hsd*) or *choD* (Δ*choD*) in the H37Rv strain background (see Supplemental Methods; ref. 32). We grew these strains in liquid cultures using either minimal or a nutrient-rich media (7H9 with oleic acid, albumin, dextrose, catalase, and glycerol), supplemented with either vehicle control or 0.1 mg/mL cholesterol solubilized in tyloxapol/ethanol (Figure 4, A and B). We monitored growth of WT and mutant strains and quantified cholesterol and cholestenone abundance. As expected, the mutants and parental strain grew similarly in nutrient-rich media, irrespective of whether it was supplemented with cholesterol (Figure 4B). In these conditions, cholesterol levels decreased over time and became undetectable by day 5. In minimal media, all the strains grew when cholesterol was provided as the sole carbon source (Figure 4A). The Δ*choD* strain was indistinguishable from WT, whereas the Δ*hsd* mutant had a slight growth delay during the exponential growth phase, accompanied by a slight delay in depleting cholesterol in the media (Figure 4A). However, despite the modest impact on cholesterol utilization, we found a drastic decrease in cholestenone production by the Δ*hsd* mutant compared with the WT strain and Δ*choD* mutant, which was restored by complementation (Figure 4C). Thus, neither ChoD nor 3β-Hsd is required for growth of *M. tuberculosis* on cholesterol as the sole carbon source, although 3β-Hsd was required for cholestenone production. The modest growth delay of Δ*hsd* was reproducible and restored by complementation (Figure 4D). Together, these results indicate that 3β-Hsd is required for cholestenone production by *M. tuberculosis* in vitro. Importantly, since the Δ*hsd* mutant is capable of growing on cholesterol even though it cannot produce cholestenone (Figure 4, A and C), our results show that cholestenone production is not absolutely essential for cholesterol utilization in *M. tuberculosis*. To determine whether 3β-Hsd was essential for cholestenone production during intracellular infection, we compared cholestenone abundance in BMDMs infected with *M. tuberculosis* and the Δ*hsd* mutant. Cholestenone was not detected in macrophages infected with the Δ*hsd* mutant, and genetic complementation restored the production of cholestenone (Figure 4E). In BMDMs that were pretreated with IFN-γ, cholestenone made during *M. tuberculosis* infection was also dependent on the bacterial 3β-Hsd (Supplemental Figure 6E). The same was found in PMA-differentiated THP-1 macrophages (Figure 4F). The dependence of cholestenone on bacterial 3β-Hsd was not due to attenuation of the mutant strain, as intracellular uptake and growth of Δ*hsd* was similar to WT *M. tuberculosis* (Supplemental Figure 6, F and G). Last, to determine whether nontuberculous mycobacterium (NTM) also generated cholestenone during infection, we infected BMDMs with *Mycobacterium abscessus*, an important cause of pulmonary disease, particularly in patients with cystic fibrosis. As for *M. tuberculosis*, we found that in macrophages infected with *M*. *abscessus*, host cholesterol was oxidized to cholestenone (Figure 4G). To conclude, we confirmed that 3β-Hsd is required for the oxidation of cholesterol into cholestenone by *M. tuberculosis*; however, 3β-Hsd is not required for cholesterol utilization by *M. tuberculosis*. In addition, cholestenone is also produced during macrophage infection with *M*. *abscessus*, consistent with the presence of cholesterol oxidases in mycobacterial species beyond *M. tuberculosis* (33).

### Cholestenone levels are elevated in clinical samples from patients with TB.

**Figure 5:**
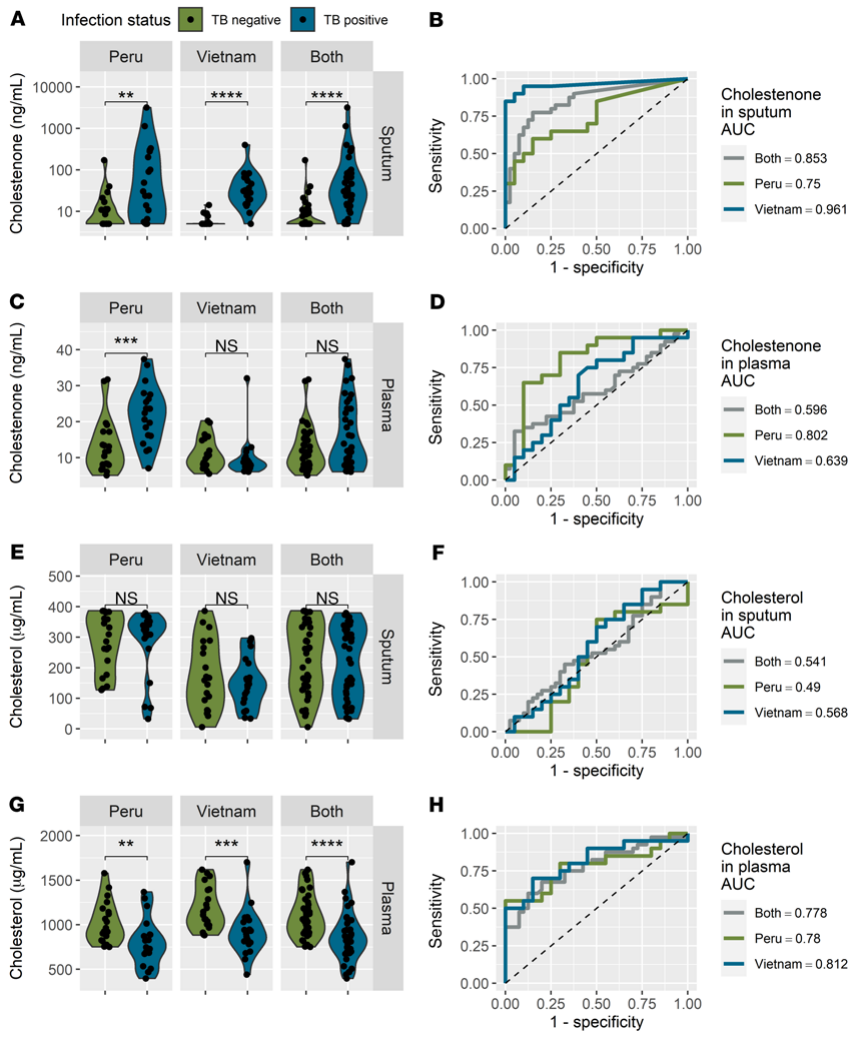
Cholestenone levels are elevated in clinical samples from TB patients. Levels of metabolites from clinical samples from TB-negative (*n =* 20 per country) and TB-positive subjects (*n =* 20 per country). (**A**) Sputum cholestenone level and (**B**) corresponding ROC curve. (**C**) Plasma cholestenone level and (**D**) corresponding ROC curve. (**E**) Cholesterol level in sputum and (**F**) corresponding ROC curves. (**G**) Cholesterol level in plasma and (**H**) corresponding ROC curves. Statistical analyses were done using the Mann-Whitney test for sputum samples and Student’s *t* tests for plasma samples. **P < 0.01, ***P < 0.001, ****P < 0.0001. The methods used for calculating cholestenone concentrations are detailed in the Supplemental Material. Metabolite abundance is reported in unprocessed samples (plasma or nonliquefied sputum).

**Figure 6:**
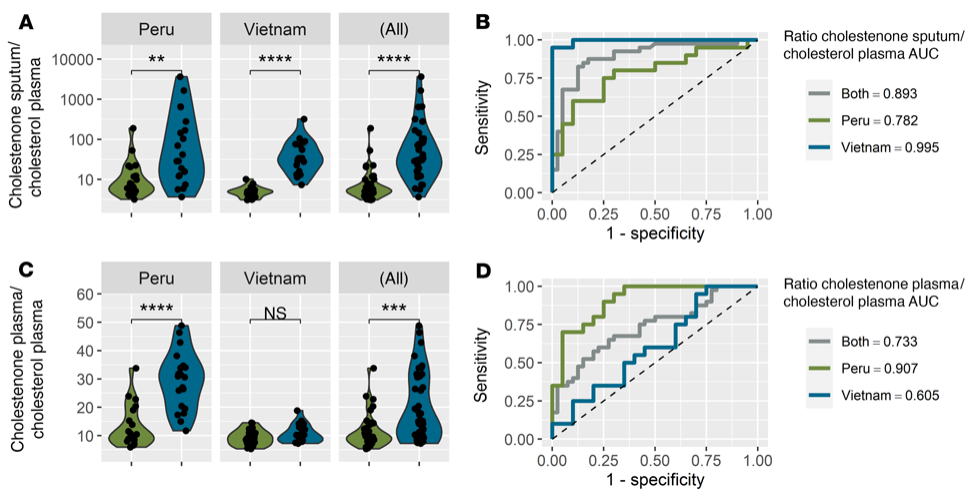
Ratio of cholestenone to plasma cholesterol in clinical samples from TB patients improves the diagnostic accuracy. Ratios of metabolites from clinical samples from TB-negative (*n =* 20 per country) and TB-positive subjects (*n =* 20 per country). (**A**) Ratio of sputum cholestenone and plasma cholesterol of control and TB-positive subjects from both countries and the (**B**) corresponding ROC curve. (**C**) Ratio of plasma cholestenone and plasma cholesterol in control patients and patients with TB from both countries, and the (**D**) corresponding ROC curve. (**A** and **C**) The ratios were determined on a per-subject basis and were multiplied by a factor 10^6^ for clarity. Statistical analyses were done using the Mann-Whitney test for sputum samples and Student’s *t* tests for plasma samples. **P < 0.01, ***P < 0.001, ****P < 0.0001. The methods used for calculating cholestenone concentrations are detailed in the Supplemental Material.

Based on our in vitro results, we investigated whether methylsuccinate, cholestenone, and itaconate are more abundant in patients with TB compared with controls. We obtained paired sputum and plasma samples from 80 patients from Peru and Vietnam. All patients were at least 18 years old and HIV negative. The TB-positive group (*n =* 20 per country) were positive by sputum smear microscopy and culture. Importantly, subjects in the control group (*n =* 20 per country) had symptoms compatible with TB infection, but they were negative for active TB by sputum smear microscopy, culture, and Xpert, as well as having negative Quanti-FERON assay results. There were no significant differences in the age range or sex of TB-positive or -negative subjects in either country (Table 1). Their clinical symptoms, such as chest pain, recent weight loss, fever, dyspnea, and hemoptysis were recorded (Table 1 and Supplemental Tables 2 and 3). Differences between the Peruvian and Vietnamese populations are shown in Supplemental Table 2. We extracted metabolites from sputum and plasma samples and measured methylsuccinate, cholestenone, and itaconate levels by mass spectrometry. Since itaconate is produced as a general host response to infection, we thought it might be present in both TB-infected and uninfected subjects, since the TB-negative subjects might have other infections. However, in most subjects, itaconate was undetectable, and we found no significant differences in levels of itaconate or methylsuccinate in sputum or plasma of TB-positive subjects compared with controls (Supplemental Figure 8). We anticipated that cholestenone would be TB-specific, since we could not find any evidence that it was made by common pulmonary pathogens. Indeed, the sputum of TB-positive subjects had significantly higher cholestenone abundance compared with controls, in both the Peru and Vietnam cohorts (Figure 5A, Mann-Whitney *P <* 0.01 and *P <* 0.0001 respectively). To assess the precision of the mass spectrometry quantification, we repeated the sputum processing and mass spectrometry analysis for 80 samples and found a high degree of correlation between the independent experiments (Supplemental Figure 7B). Receiver operating characteristic (ROC) analysis showed that the levels of cholestenone in sputum had high TB diagnostic potential in Vietnam (AUC = 0.96), and Peru (AUC = 0.75; Figure 5B). In addition to higher levels of cholestenone in sputum, patients with TB in the Peru cohort also had significantly higher plasma cholestenone compared with controls (Figure 5C, *t* test *P <* 0.001). Remarkably, plasma levels of cholestenone were predictive of TB infection in subjects from Peru (Figure 5D; AUC = 0.80). However, plasma cholestenone levels were not significantly associated with TB infection status in the Vietnam cohort and showed poor predictive value (Figure 5, C and D; AUC = 0.64). While sputum cholestenone levels were elevated in subjects with TB, sputum cholesterol levels were not different between TB-infected and control subjects and were therefore of little predictive value (Figure 5, E and F). Plasma cholesterol levels in both cohorts were significant lower in subjects with TB compared with controls (Figure 5G, *t* test *P <* 0.01 and *P <* 0.001 for Peru and Vietnam cohorts, respectively). Consistent with these observations, ROC plots for cholesterol abundance in plasma show a similar performance for both cohorts (Figure 5H, AUC = 0.81 in Vietnam and AUC = 0.78 in Peru). Absolute quantification values and statistical parameters for cholestenone and cholesterol concentrations in these samples are reported in Supplemental Table 4. Furthermore, a ratio of sputum cholestenone and plasma cholesterol was significantly increased in TB-positive subjects compared with controls in both countries, and using the ratio improved the discriminating potential of these metabolites (Figure 6, A and B; AUC = 0.995 in Vietnam cohort, AUC = 0.78 in Peru cohort). Consistent with the observations for cholestenone plasma levels in TB-positive subjects from Peru, the ratio of plasma cholestenone and plasma cholesterol was significantly higher in TB-positive subjects from Peru than in controls and had a high diagnostic accuracy (Figure 6, C and D, AUC = 0.90 in Peru). To conclude, we found that bacterial-derived cholestenone, which is a dominant metabolic signature of *M. tuberculosis* infection in macrophages, is elevated in samples from TB-infected subjects in a clinical setting.

### Cholestenone abundance in sputum of patients with TB correlates with infection burden.

**Figure 7:**
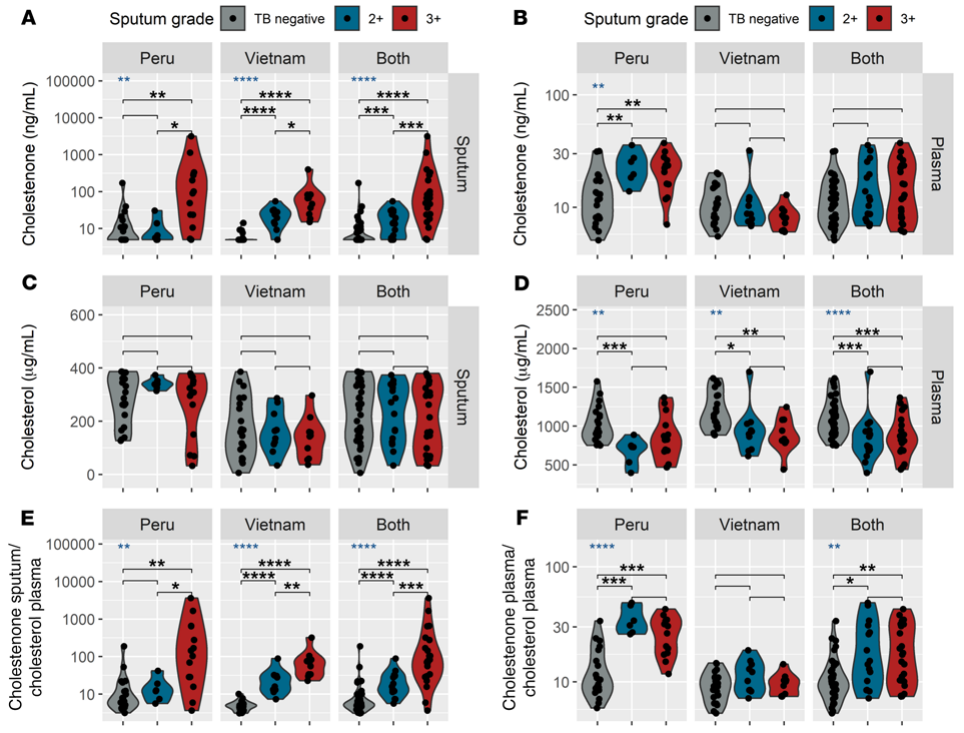
Cholestenone abundance in sputum of patients with TB correlates with infection burden. Levels of metabolites are plotted for TB-negative subjects and TB-positive subjects grouped according to sputum grade, for (**A**) cholestenone in sputum, (**B**) cholestenone in plasma, (**C**) cholesterol in sputum, (**D**) cholesterol in plasma, (**E**) the ratio of cholestenone in sputum divided by cholesterol in plasma, (**F**) the ratio of cholestenone in plasma divided by cholesterol in plasma. Results are shown for each country (*n =* 40) as well as both countries pooled (*n =* 80). The ratios were determined on a per-subject basis and are shown multiplied by a factor 10^6^ for clarity. Statistical analyses were done using the Kruskal-Wallis test (blue asterisks) followed by pairwise comparisons with the Mann-Whitney test with the Benjamini-Hochberg correction for multiple comparisons. The resulting adjusted *P* values are shown with black asterisks. For all statistical tests, significance is indicated with the following: *P < 0.05, **P < 0.01, ***P < 0.001, ****P < 0.0001. Lack of asterisks indicates *P* > 0.05. The methods used for calculating cholestenone concentrations are detailed in the Supplemental Material. Metabolite abundance is reported in unprocessed samples (plasma or nonliquefied sputum).

Next, we assessed whether clinical characteristics of the TB-positive subjects influenced cholestenone and cholesterol levels in sputum or plasma (Supplemental Table 3). First, we grouped TB-positive patients from both Peru and Vietnam (*n =* 40) according to sputum grade (2+ or 3+), and compared each group with TB-negative subjects (*n =* 40; Figure 7). We found that the cholestenone levels in sputum were higher for the 2+ subjects compared with TB-negative subjects (Figure 7A, adjusted *P* < 0.01), and a further increase was seen in sputum of the 3+ subjects compared with 2+ subjects (Figure 7A, adjusted *P* < 0.001), suggesting that sputum cholestenone abundance correlates with *M. tuberculosis* burden in the lungs. In contrast, there was no difference in plasma cholestenone levels based on sputum grade (Figure 7B). Cholesterol levels in sputum were also not different between subjects based on sputum grade (Figure 7C). Plasma cholesterol levels were decreased in 2+ TB-positive and 3+ TB-positive subjects compared with TB-negative subjects, however there was no difference between 2+ and 3+ subjects (Figure 7D). Next, we grouped the TB-positive subjects from both countries (*n =* 40) and investigated whether metabolite levels were associated with other clinical parameters such the subject’s sex or presence of specific clinical symptoms (Supplemental Table 3). We found no clinical parameter that was significantly associated with cholestenone or cholesterol levels in the sputum of TB-positive subjects (Supplemental Table 3). Plasma cholesterol, however, was significantly lower in patients with hemoptysis, a sign of advanced disease (Supplemental Table 3; *t* test *P <* 0.05). To conclude, we show that sputum cholestenone level in patients with TB correlates with the degree of smear positivity, suggesting it reflects pulmonary pathogen burden.

## Discussion

In the present study, we interrogated the metabolic landscape of *M. tuberculosis*–infected macrophages. We found that the host recalibrates its metabolism to support immune effector functions. *M. tuberculosis* infection induces a Warburg-like glycolytic shift, enhances flux to the pentose phosphate pathway, and modifies the TCA cycle for generating the antimycobacterial metabolite itaconate, the top fourth differential biochemical in our screen. This intracellular milieu poses a challenge for most microbes, but *M. tuberculosis* is highly adapted to survive this environment. We found that the most prominent metabolic signature of *M. tuberculosis* infection is likely to reflect bacterial cholesterol degradation. Bacterial cholesterol metabolism results in propionyl-CoA, which is metabolized through the methyl citrate and methylmalonyl pathway, and propionyl-CoA, 2-methylcitrate, and methylmalonate are infection-induced metabolites. In addition, previous work has shown that growing *M. tuberculosis* in the presence of cholesterol results in elevated levels of methylsuccinate and 2-aminoadipate (20), also top differential metabolites in our study. We cannot infer anything about the abundance of other metabolites of cholesterol degradation by *M. tuberculosis*, such as ADD, 9OHADD, DSHA, or 3-HSA (21), because they were not included in the panel of queried metabolites in the screening platform. Since humans do not degrade cholesterol through a similar pathway, this raised the possibility that unique cholesterol metabolites, such as cholestenone, might be biomarkers for infection. Indeed, we found that the oxidized cholesterol metabolite cholestenone accumulates in *M. tuberculosis*–infected macrophages, and cholestenone levels in sputum correlate with active TB disease in 2 geographically distinct cohorts. While cholesterol utilization by *M. tuberculosis* has been linked to dormancy and persistence (8, 15), our data suggest that *M. tuberculosis* metabolizes cholesterol during active infection in people, consistent with expression profiling showing activation of the KstR regulon in *M. tuberculosis* isolated from sputum (34). Our data in macrophages suggest that intracellular *M. tuberculosis* has access to host cholesterol, and we anticipate that extracellular bacilli would also be able to metabolize cholesterol present extracellularly in caseum or sputum.

Our data suggest that the vast majority of cholestenone present during *M. tuberculosis* infection is derived by host-pathogen cometabolism, with the host providing cholesterol and the *M. tuberculosis* 3β-Hsd enzyme converting it to cholestenone. What is the evidence in support of this idea? First, recombinant *M. tuberculosis* 3β-Hsd has been shown to convert cholesterol to cholestenone in vitro (29, 35). *M. tuberculosis* lacking Rv1106c, the gene encoding 3β-Hsd, fail to convert cholesterol to cholestenone when growing in liquid culture, as shown by others (29) and verified by us (Figure 4C). We found that in macrophages the abundance of cholestenone that accumulates upon *M. tuberculosis* infection depends upon duration and MOI (Figure 3B). Importantly, cholestenone production in macrophages also depends on the bacteria being alive and having the *hsd* gene (Figure 3B, Figure 4, E and F, and Supplemental Figure 6E). Thus, while it is theoretically possible that the cholestenone produced in macrophages is made by a host enzyme in response to WT but not Δ*hsd* mutant bacilli, the most straightforward explanation is that the cholestenone made during infection comes from 3β-Hsd of the bacilli. In addition, although humans have several 3β-HSD enzymes that bear 29% to 35% identity to the one in *M. tuberculosis*, as far as we are aware, no mammalian enzyme is reported to convert cholesterol to cholestenone. When we examined available published data sets, we found that the mammalian 3β-hydroxysteroid dehydrogenases (HSD3B1 and HSD3B2) do not appear to be expressed in *M. tuberculosis*–infected murine macrophages, *M. tuberculosis*–infected human macrophages, and IFN-γ activated murine macrophages (36–38). This is not surprising since they are expressed in steroidogenic tissue where they dehydrogenate steroid hormones. Therefore, all the data combined suggest that *M. tuberculosis* 3β-Hsd converts cholesterol to cholestenone during macrophage infection.

Although the evidence strongly suggest that *M. tuberculosis* is the source of cholestenone during macrophage infections, how is it then that we detected low levels of cholestenone in plasma of subjects without TB? These subjects had plasma cholestenone levels in the range of 5 to 32 ng/mL (Supplemental Table 4), consistent with a previous report that demonstrated levels of 30.4 ± 8.5 ng/mL from a pooled sample of plasma from 100 individuals representative of the US population (39). HSD3B1 and HSD3B2 catalyze the conversion of 3β-hydroxy steroids, which lack the side chain found on cholesterol, to the 3-keto configuration. They are expressed in the adrenals, ovaries, and testes, where they carry out an essential step in production of progesterone, androstenedione, and testosterone. HSD3B7 converts the 3β-hydroxy of 7α-hydroxycholesterol to the 3-keto configuration during bile acid synthesis. Cholestenone is not reported as an intermediate in either of these pathways. Thus, the source of the baseline cholestenone in the subjects without TB is not clear, but 2 recent publications suggest that it may be derived from cholesterol dehydrogenases expressed from uncultured members of the microbiome (40, 41). Thus, while our findings support the idea that in TB-infected subjects the elevated cholestenone is largely *M. tuberculosis*–derived, it could theoretically be microbiome-derived or the result of a yet-to-be-defined host enzyme.

We found that although 3β-Hsd is required for cholestenone production, it is not required for cholesterol utilization, at least in the H37Rv strain background. This was somewhat surprising because cholesterol oxidation to cholestenone is often considered an initial event in cholesterol degradation. Cholesterol is degraded through β-oxidation of the cholesterol side chain and cleavage of the A and B rings, followed by degradation of the C and D rings (21). Side chain degradation is initiated by oxidation of the side chain at C26. In H37Rv, CYP125 and CYP142 can both perform this initiating step, and since both cholesterol and cholestenone are known substrates of CYP125 and CYP142 (42), it is not surprising that this could occur without 3β-Hsd. However, the first enzyme involved in AB ring cleavage, KstD (Rv3537), strongly prefers androstendione (AD) as a substrate, which has a ketone at the third carbon, compared with a substrate with a 3-hydroxy (43). It is possible that in the absence of 3β-Hsd, a different enzyme acts during or after cholesterol side chain degradation, for example, by converting the 3-hydroxy of dehydroepiandrosterone to the 3-ketone of AD. Brzostek et al showed that the Δ*hsd* Δ*choD* double mutant in the H37Rv strain background makes AD and 9-hydroxy androstendione (9OHAD), consistent with the idea that there is an enzyme other than 3β-Hsd and ChoD that can generate the ketone. It is also possible that there is metabolic flexibility and a ketone at C3 is not absolutely essential for *M. tuberculosis* to grow on cholesterol. To understand differences in how cholesterol and cholestenone are degraded in the absence of 3β-Hsd will require dedicated studies with labeled cholesterol and cholestenone. It should be pointed out that our study is in agreement with 2 previous reports on the role of 3β-Hsd in cholesterol degradation and may resolve why they were seemingly at odds with one another (29, 31). As reported by Yang et al. (29), we validated that *M. tuberculosis* 3β-Hsd is the major cholesterol-oxidizing enzyme that generates cholestenone, whereas ChoD appears to be dispensable for this activity (Figure 4C). As reported by Brzostek et al. (31), 3β-Hsd is not required for utilization of cholesterol. Thus, the apparent discrepancy between these previous studies is explained by the finding that the ability of *M. tuberculosis* to grow on cholesterol does not depend on the oxidation of cholesterol to cholestenone.

Previous studies have shown that cholestenone disrupts lipid rafts, membrane fluidity, and cell signaling in mammalian cells (44–48), and recent work shows that cholestenone has antimicrobial activity against *Helicobacter pylori* (49). We found that during macrophage infections cholestenone appears to accumulate over time. Whether cholestenone remains associated with the bacilli or traffics to host membranes is an area for future investigation. Given that it is detected in both sputum and plasma in clinical samples, it seems likely that it is widely distributed and could have a biological impact. Since 3β-Hsd can also oxidize oxysterols, such as pregnenolone (29), *M. tuberculosis* might also modify steroid hormones. Interestingly, *Mycobacterium leprae* has lost the genes for cholesterol catabolism, but retains *hsd* and the ability to make cholestenone (50, 51). In addition, in *M. tuberculosis* Rv1106c/*hsd* is not transcriptionally regulated with other genes required for cholesterol metabolism (52). Combined, these observations suggest that cholestenone or other 3β-Hsd–generated host-pathogen cometabolites might be involved in pathogenesis. As described for tryptophan cometabolism (53), our work suggests that utilization of host cholesterol by *M. tuberculosis* has a role beyond bacterial nutrition.

Cholestenone does not appear to be a metabolite made by most pulmonary pathogens either. The literature suggests that cholestenone can be produced by a limited number of microbes that infect humans including *Mycobacteria*, *Rhodococcus,* and *Nocardia* (54). In addition, we performed a literature review on the top bacterial causes of pulmonary infections and only identified reports of cholesterol oxidase activity by ChoD orthologs in *Acinetobacter* (55), *Pseudomonas* (56–58), and *Serratia* (59). However, there are several studies showing that putative cholesterol oxidases of *Pseudomonas* produce hydroperoxycholestenone (HCEO) rather than cholestenone (56–58). In addition, outside of *Mycobacteria*, there are very little data available on 3β-Hsd proteins in prokaryotes. Two recent publications report 3β-Hsd orthologs in bacterial species of the human gut microbiota (40, 41). To our knowledge, no other reports describe 3β-Hsd family proteins in bacterial species outside of *Mycobacteria*. Thus, we hypothesized that cholestenone might be a specific differential diagnosis biomarker of active TB disease, generated by the cometabolism of host and pathogen. Indeed, we found that cholestenone levels in sputum correlated with TB infection status in human patients. In our study, the control group consisted of subjects presenting with symptoms consistent with TB, but in whom *M. tuberculosis* infection was ruled out. This suggests that the significantly elevated level of cholestenone in the TB-positive group is specific to *M. tuberculosis* infection rather than a more general marker of lung infection or inflammation. We also observed hypocholesterolemia in TB patient plasma in both Peru and Vietnam, a finding previously documented in populations in Turkey and Ethiopia (60, 61). While there are many reasons this might be, it is tempting to speculate that consumption of cholesterol by *M. tuberculosis* contributes to reduced levels in the host. Taken together, a ratio of sputum cholestenone and plasma cholesterol showed excellent predictive accuracy for diagnosing TB in both Peru and Vietnam cohorts.

Interestingly, we found that plasma cholestenone levels also correlated with TB infection status, but only in the Peruvian population. In sputum, the TB diagnostic potential of cholestenone was higher in subjects from Vietnam (AUC = 0.96) than in those from Peru (AUC = 0.75). What might account for these differences? Cholestenone might be metabolized differently in the 2 populations because of differences in bacterial strains or host genetics. Since different lineages of *M. tuberculosis* predominate in Peru and Vietnam (62), there might be differences in how the bacilli metabolize cholesterol, which could contribute to differences in metabolites detected in people. Hsd is uniformly present in *M*. *tuberculosis* isolates, but it might be regulated differently or other cholesterol metabolic genes may vary by lineage. Since different lineages have also been shown to have different predilection for extra-pulmonary disease manifestations, the plasma levels may reflect these differences. Our knowledge of how cholestenone is metabolized in humans is extremely limited. For example, if the Vietnamese cohort hydroxylates cholestenone or otherwise modifies it in plasma, we would not detect the modified species. In addition, differences in metabolism by gut microbiota might impact metabolite levels in the different human populations (40, 41). Studies in rats and patients with cerebrotendinous xanthomatosis show that cholestenone can be converted into cholestanol in the liver (63, 64). In addition, alveolar macrophages express CYP27A1, a sterol 27-hydroxylase that converts cholesterol to cholestenoic acid (65). CYP27A1 hydroxylates cholestenone at a much higher rate than it modifies cholesterol (66). Thus, conversion of cholestenone to 27-hydroxycholestenone or oxidation of 25-hydroxylated metabolites of cholesterol that are induced by interferon signaling might vary between the Peruvian and Vietnamese populations. Thus, future investigation to understand the basis of the differences between the 2 populations will be important and might identify cholestenone-related metabolites that would be superior TB biomarkers in both sputum and plasma. While the performance of cholestenone in sputum would fall short of the WHO target product profile for a TB disease biomarker in Peru, evaluating additional cholestenone-related species may lead to a superior test. A non-sputum-based diagnostic is a high priority for TB diagnostics (67). In Peru, we found that the ratio of cholestenone to cholesterol in plasma samples performed extremely well as a diagnostic for active TB (AUC = 0.90). It will be critical to see if this can be improved upon and adapted to other populations.

We also found that cholestenone levels in sputum correlated with the degree of smear positivity. If sputum cholestenone reflects disease burden, then sputum levels might be used to monitor treatment. One limitation of our study is that smear positivity is an imperfect measure of disease burden, and all of our subjects had a high level of smear positivity. It will be important to follow cholestenone levels in patients with TB longitudinally to determine whether it declines with therapy, or can predict cure or relapse. It will also be important to evaluate cholestenone levels in patients with latent TB, extrapulmonary TB, smear-negative disease, and with comorbidities such as HIV infection and diabetes. Although a mass spectrometry–based assay of *M. tuberculosis* disease burden would be useful in research settings, to be a useful clinical tool in resource-constrained, high-burden countries will require translating it into a point-of-care test.

Homologs of 3β-Hsd have been annotated in the genomes of most NTM species (33), and we found that *M*. *abscessus* also produces cholestenone during infection. Thus, cholestenone might be a useful biomarker for NTM infections as well. While TB incidence has declined in the United States, infections with NTM are rising (68). NTM infections require extremely long courses of treatment, usually more than a year, and they are often more challenging to treat than *M. tuberculosis* because of limited antibiotic options. Biomarkers that could guide clinical decision making would be extremely useful. While isolation of *M. tuberculosis* from a sputum sample is always diagnostic of TB infection, isolation of NTM can occur because of environmental contamination. Detection of cholestenone could be a rapid way to distinguish true infection from contamination. In addition, a mass spectrometry–based test is feasible in clinical microbiology laboratories in high income settings where NTM infections are an increasing problem.

We show that cholestenone is a prominent metabolic signature of mycobacterial infections, and *M. tuberculosis* uses the cholesterol oxidase 3β-Hsd to produce it. While there are a number of *M. tuberculosis*–derived molecules that have been pursued for diagnostic and biomarker purposes (2), as far as we are aware, cholestenone is unique in being the result of active bacterial-host cometabolism. Recent efforts to develop novel diagnostics and biomarkers have focused on detecting a distinctive signature of the host response to TB infection (69). One challenge for host-based diagnostics is that with clinical use, coinfections and comorbidities impact the host signatures. Thus, to date there has been considerable progress identifying TB biomarkers, but efforts continue to be hampered by paucibacillary disease and heterogeneity of the host response. Our findings indicate that cholestenone and related cometabolites should be further evaluated for their role in pathogenesis and as potential TB biomarkers.

## Methods

### Bacterial strains.

*M*. *tuberculosis* (H37Rv strain) was grown aerobically at 37°C in Middlebrook 7H9 broth with 0.05% Tyloxapol, 0.2 % glycerol, and OADC (oleic acid-albumin-dextrose-catalase; catalog 212351; BD Biosciences). When required, antibiotics were added in the culture media with the following concentrations: 25 μg/mL kanamycin, 50 μg/mL hygromycin, and 25 μg/mL zeocin. The strains used in this study, details for their construction, and primers used can be found in the Supplemental Methods and Supplemental Tables 5 and 6. The mutant strains are isogenic derivatives of H37Rv, built using ORBIT (Oligonucleotide-mediated Recombineering followed by Bxb1 Integrase Targeting) mediated mutagenesis (32) and confirmed by PCR analysis. The Δ*hsd* mutant was complemented with an integrating plasmid (pCH89), containing the full Hsd operon (Rv1106c- Rv1109c). PDIM production by the strains was confirmed by mass spectrometry. Details of plasmid construction can be found in Supplemental Methods. The pKM464 and pKM461 plasmids were gifts from Kenan Murphy and Chris Sassetti (University of Massachusetts Medical School).

### Cell culture.

Bone marrow–derived macrophages (BMDMs) were obtained from 8- to 12-week-old C57Bl/6 mice that were obtained from The Jackson Laboratory. Bone marrow was flushed from the femurs and tibia of mice as described (4). BMDMs and THP-1 cells (American Type Tissue Collection) were maintained as previously described (4). To promote macrophage differentiation, THP-1 cells were treated with 100 nM phorbol myristate acetate (PMA; Sigma) for 24 hours before infection. BMDMs were activated with IFN-γ (Thermo Fisher Scientific, catalog PMC4031) at a concentration of 100 U/mL.

### Global metabolomics profiling.

For global metabolic profiling, BMDMs isolated from 8 C57Bl/6 mice (aged 8–12 weeks obtained from The Jackson Laboratory) were pooled and used for 4 experimental groups: uninfected and *M. tuberculosis*–infected, at 3 and 24 hour time points. Each group had 5 replicates (*n =* 20 samples total). Fifteen million murine BMDMs were infected with H37Rv at an MOI of 5 for 4 hours. Cells were then washed to remove extracellular bacteria and maintained in culture medium for 3 or 24 hours. At the respective time points, BMDMs were washed twice with sterile Hank’s Balanced Salt Solution (HBSS, Gibco), and metabolites were extracted in 80% methanol (Sigma) in water (Corning) containing premeasured internal standards provided by Metabolon. The samples were stored at –80°C prior to shipping to Metabolon for further processing and analyses. At Metabolon, the samples were prepared using the automated MicroLab STAR system from Hamilton Company. After addition of recovery standards and protein removal, the extracts were divided into fractions for analysis as follows: 2 separate reverse phase (RP)/ultra-performance liquid chromatography mass spectometry (UPLC-MS)/MS with positive ion mode electrospray ionization (ESI), RP/UPLC-MS/MS with negative ion mode ESI, and hydrophilic interaction liquid chromatography (HILIC)/UPLC-MS/MS with negative ion mode ESI. All methods utilized a Waters Acquity UPLC and a Thermo Scientific Q-Exactive high resolution/accurate mass spectrometer interfaced with a heated electrospray ionization (HESI-II) source and Orbitrap mass analyzer operated at 35,000 mass resolution. Raw data were extracted, peak-identified, and QC processed using Metabolon’s hardware and software that are built on a web-service platform using Microsoft’s NET technologies. Metabolon maintains a library based on authenticated standards that contain the retention time/index (RI), mass-to-charge ratio, and chromatographic data (including MS/MS spectral data) on all molecules present in the library. To distinguish biochemicals, their identifications were based on 3 criteria: retention index within a narrow RI window of the proposed identification, accurate mass match to the library ± 10 ppm, and the MS/MS forward and reverse scores between the experimental data and authentic standards. Peaks were quantified using AUC. Missing values were imputed with the minimum observed value for each compound. To determine scaled intensity, the raw area counts for each biochemical were rescaled to set the median equal to 1. Statistical analyses were conducted on the scaled and imputed data. Refer to Supplemental Methods for additional details.

### Random forest analysis.

Random forest represents a supervised classification technique based on biochemical profile of the data set. Random forest analysis was done in RStudio using the package ‘randomForest’ with sampsize = rep(m1, l1) and ntree = 50,000, where m1 is half the smallest group in the random forest, l1 is the number of groups, and ntree is the number of trees. To build the random forest, all 507 metabolites were included from 19 of 20 samples (one replicate was considered an outlier; see Supplemental Figure 1). The analysis misidentified 1 infected sample resulting in an out-of-bag (OOB) error of 1/19 and predictive accuracy of 95%.

### In vitro growth assays.

To assess *M. tuberculosis* growth in defined carbon sources, the strains were grown in minimal media (0.5 g/L asparagine, 1 g/L KH_2_PO_4_, 2.5 g/L Na_2_HPO_4_, 5 mg/L ferric ammonium citrate, 0.5 g/L MgSO_4_-7H_2_O, 0.5 mg/L CaCl_2_, and 0.1 mg/L ZnSO_4_), adapted from ref. 8. As needed, carbon sources were provided either through 0.1 % glycerol (vol/vol) or 0.01 % cholesterol (wt/vol). Cholesterol was added using a 100× stock solution prepared in tyloxapol/ethanol (1:1). Growth was monitored using a cell density meter and normalized to the optical density of the culture medium. Throughout the growth curves, bacterial samples were withdrawn for cholesterol and cholestenone quantification. For cholesterol quantification, at defined time points, culture samples were frozen at –80°C, until they were processed together for total cholesterol (free cholesterol + esterified cholesterol) quantification using the Amplex Red Cholesterol Assay Kit (Invitrogen). For cholestenone analysis, the bacterial culture samples were centrifugated, washed twice in PBS, and the pellets stored at –80°C until they were processed for mass spectrometry. For the investigation of cholestenone bactericidal activity on *M*. *tuberculosis*, cholestenone was prepared in ethanol as 100× stock solutions to achieve indicated final concentrations.

### Chemicals and reagents.

The following compounds were used as internal standards or reference compounds: (±)-2-methyl-d3-succinic-2,3,3-d_3_ acid (catalog M329046; Toronto Research Chemicals [TRC]), methylmalonic acid-d_3_ (catalog M318862; TRC), 2-methylcitric acid-d_3_ (catalog M265082; TRC), itaconic acid-^13^C5 (catalog I931004; TRC), 4-cholesten-3-one-2,2,4,6,6-d5 (catalog D-5467; CDN Isotopes) 4-cholesten-3-one (catalog 188174; Sigma-Aldrich), itaconic acid (catalog I29204; Sigma-Aldrich), methylcitric acid (catalog M265080; TRC), methylmalonic acid (catalog M318862; TRC), and methylsuccinic acid (catalog M329045: TRC). All HPLC grade solvents were purchased from Sigma.

### Mass spectrometry identification of metabolites.

Liquid chromatography-tandem mass spectrometry (LC-MS/MS) methods were used to analyze metabolites extracted from samples derived from either bacterial pellets, infected cells, or human clinical samples obtained from the Foundation for Innovative New Diagnostics (FIND). Samples were extracted with 80% methanol, and the appropriate internal standards were included in the extraction buffer at final concentrations: 0.20 μg/mL 4-cholesten-3-one-2,2,4,6,6-d5, 0.1 μg/mL (±)-2-methyl-succinic-2,3,3-d_3_ acid, 1 μg/mL itaconic acid-^13^C5, 0.1 μg/mL methylmalonic acid-d_3_, or 0.1 μg/mL 2-methylcitric acid-d_3_.

All samples were extracted with 80% methanol for 10 minutes at room temperature, followed by centrifugation for 10 minutes at 21,130*g*, and the supernatants were sent to the Washington University Metabolomics Facility for LC-MS/MS analysis. Bacterial pellets from in vitro growth were resuspended in 500 μL 80% methanol. For samples obtained from infected macrophages, 1 × 10^6^ cells were infected, and at defined time points they were washed twice in PBS and extracted with 500 μL 80% methanol. Sputum samples were liquefied before methanol extraction by adding Sputolysin (Calbiochem) in a 1:1 proportion. Plasma and sputum samples were added to 100% methanol to achieve a volume of 500 μL extraction buffer at a final concentration of 80% methanol. The measurement of 4-cholesten-3-one, methylsuccinic acid, itaconic acid was performed with a Shimadzu 20AD HPLC system (Shimadzu) coupled to a Q Exactive mass spectrometer or 4000QTRAP mass spectrometer (AB Sciex). The 4-cholesten-3-one was separated on an ACE C8 (4.6 × 100 mm, 3 μm) column (Mac-Mod Analytical) and detected with positive multiple reaction monitoring (MRM) mode. The methylsuccinic acid and itaconic acid were separated on a ZIC-HILIC (4.6 × 150 mm, 3 μm) column and detected with negative MRM mode. Quality control samples were prepared by pooling a portion of the study samples, and were injected after every 10 study samples to monitor instrument performance. Data processing was conducted with Analyst 1.6.3. The methods to calculate cholestenone concentration are detailed in Supplemental Methods. Metabolite abundance is reported for unprocessed samples (plasma or nonliquefied sputum).

### Bacterial infections.

For in vitro macrophage assays, a log-phase culture of *M. tuberculosis* was pelleted and resuspended in macrophage culture medium. Bacterial single-cell suspensions were prepared by low speed centrifugation (60*g* for 8 minutes). The number of *M. tuberculosis* in the resulting supernatant was estimated by measuring absorbance at 600 nm, followed by infection of macrophages at a MOI of 1, 5, or 10. The infectious dose administered was calculated by plating CFUs from an aliquot of the bacterial suspension. After 4 hours, macrophages were washed 3 times with warm media to remove extracellular bacteria. To estimate intracellular *M. tuberculosis* growth, infected macrophages were lysed in 0.06% sodium dodecylsulfate (SDS) solution at the indicated time points, and serial dilutions of the lysates were plated on 7H10 agar plates (catalog 283810; BD Biosciences) containing glycerol and Middlebrook OADC enrichment (oleic acid-albumin-dextrose-catalase, catalog 212351; BD Biosciences). The number of CFUs was calculated 14 to 21 days later.

### Clinical study design.

The goal of the study was to determine whether cholestenone, cholesterol, methylsuccinate, or itaconate levels were different in TB-positive subjects compared with TB-negative controls. We obtained paired sputum and plasma samples from 20 TB-positive and 20 TB-negative subjects from Peru and Vietnam from FIND (https://www.finddx.org/specimen-bank/specimens-tb/). The TB-negative samples were from subjects who tested negative on sputum smear, culture, Xpert, and Quantiferon testing. These subjects had symptoms consistent with TB, but their symptoms improved or resolved at follow-up without treatment. The TB-positive samples were from sputum smear, culture, and Xpert-positive subjects who had symptoms consistent with *M. tuberculosis* infection. All patients were adults (≥ 18 years) and confirmed HIV negative. The study was exploratory, and no power analysis was performed. We requested 160 samples from 80 subjects from 2 different geographic areas. The investigators who processed the samples and quantified the metabolites were blinded to sample identity. No outliers were excluded. Total cholesterol was estimated in the plasma and liquefied sputum samples using the Amplex Red Cholesterol Assay kit.

### Statistics.

Violin plots and ROC plots were generated with RStudio version 1.3.1073 (70) using packages described in the Supplemental Methods. For the global metabolomic study, analysis of variance (ANOVA) was performed for all analytes identified in the screen. Differences between other experimental groups were assessed using 2-tailed Student’s *t* test, Mann-Whitney *U* test, or 1-way ANOVA models followed by Tukey’s post hoc tests, as indicated in figure legends. Analyses evaluating differences between TB groups and clinical characteristics included independent groups *t* tests, Mann-Whitney *U* tests, χ^2^ tests, or Fisher’s exact tests, as appropriate. A *P* value less than 0.05 was considered significant. All analyses were conducted using GraphPad Prism software (www.graphpad.com), SAS version 9.4 (SAS Institute Inc.), or RStudio version 1.3.1073. Figure 1, Figure 2 and graphical abstract were created with BioRender using original templates.

### Study approval.

The Washington University School of Medicine Institutional Animal Care and Use Committee approved all the work with mice. Euthanasia was performed in accordance with the 2020 *AVMA Guidelines for the Euthanasia of Animals*. Human clinical samples were obtained from FIND (https://www.finddx.org/specimen-bank/specimens-tb/). All specimens were collected at qualified clinics under a protocol approved by an Institutional Review Board and with informed consent from patients. FIND follows good clinical and laboratory practice in obtaining and processing samples.

## Author contributions

The project was conceptualized and experiments were designed by PC, HC, and JAP. Experiments were conducted by PC and HC and formal analysis was performed by MA, CWG, HC, PC, and JAP. Funding was acquired by PC and JAP. Writing of the original draft, review, and editing were performed by HC, PC, and JAP. JAP supervised the studies. PC and HC share the first author position. This reflects their equivalent contributions, with PC listed first based upon her seniority on the project.

## Supplementary Material

Supplemental data

Supplemental table 1

## Figures and Tables

**Table 1:**
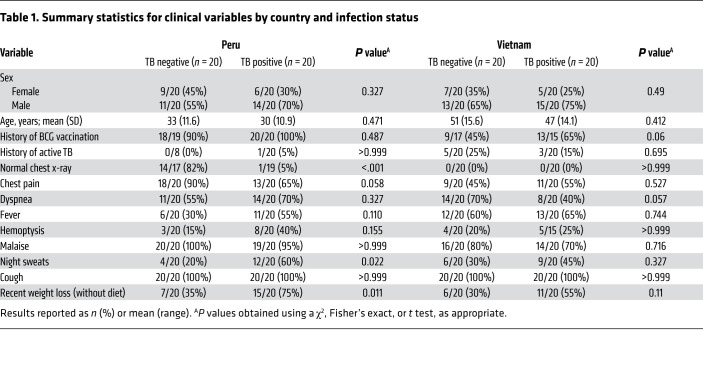
Summary statistics for clinical variables by country and infection status

## Version 1 02/01/2022

Electronic publication

## References

[2] Goletti D (2018). Update on tuberculosis biomarkers: From correlates of risk, to correlates of active disease and of cure from disease. Respirology.

[3] Ouimet M (2016). *Mycobacterium tuberculosis* induces the miR-33 locus to reprogram autophagy and host lipid metabolism. Nat Immunol.

[4] Chandra P (2020). Inhibition of fatty acid oxidation promotes macrophage control of *Mycobacterium tuberculosis*. mBio.

[5] Jha AK (2015). Network integration of parallel metabolic and transcriptional data reveals metabolic modules that regulate macrophage polarization. Immunity.

[6] Van den Bossche J (2017). Macrophage immunometabolism: where are we (going)?. Trends Immunol.

[7] Lampropoulou V (2016). Itaconate links inhibition of succinate dehydrogenase with macrophage metabolic remodeling and regulation of inflammation. Cell Metab.

[8] Pandey AK, Sassetti CM (2008). Mycobacterial persistence requires the utilization of host cholesterol. Proc Natl Acad Sci U S A.

[9] Yang X (2009). Cholesterol metabolism increases the metabolic pool of propionate in *Mycobacterium tuberculosis*. Biochemistry.

[10] Russell DG (2009). Foamy macrophages and the progression of the human tuberculosis granuloma. Nat Immunol.

[11] Peyron P (2008). Foamy macrophages from tuberculous patients’ granulomas constitute a nutrient-rich reservoir for *M. tuberculosis* persistence. PLoS Pathog.

[12] Singh V (2012). *Mycobacterium tuberculosis*-driven targeted recalibration of macrophage lipid homeostasis promotes the foamy phenotype. Cell Host Microbe.

[13] Cumming BM (2018). *Mycobacterium tuberculosis* induces decelerated bioenergetic metabolism in human macrophages. Elife.

[14] Daniel J (2011). *Mycobacterium tuberculosis* uses host triacylglycerol to accumulate lipid droplets and acquires a dormancy-like phenotype in lipid-loaded macrophages. PLoS Pathog.

[15] Soto-Ramirez MD (2017). Cholesterol plays a larger role during *Mycobacterium tuberculosis* in vitro dormancy and reactivation than previously suspected. Tuberculosis (Edinb).

[16] Wipperman MF (2014). Pathogen roid rage: cholesterol utilization by *Mycobacterium tuberculosis*. Crit Rev Biochem Mol Biol.

[17] Shi L (2015). Infection with *Mycobacterium tuberculosis* induces the Warburg effect in mouse lungs. Sci Rep.

[18] Michelucci A (2013). Immune-responsive gene 1 protein links metabolism to immunity by catalyzing itaconic acid production. Proc Natl Acad Sci U S A.

[19] Nair S (2018). *Irg1* expression in myeloid cells prevents immunopathology during *M. tuberculosis* infection. J Exp Med.

[20] Griffin JE (2012). Cholesterol catabolism by *Mycobacterium tuberculosis* requires transcriptional and metabolic adaptations. Chem Biol.

[21] Wilburn KM (2018). Cholesterol and fatty acids grease the wheels of Mycobacterium tuberculosis pathogenesis. Pathog Dis.

[22] Crowe AM (2017). Catabolism of the last two steroid rings in *Mycobacterium tuberculosis* and other bacteria. mBio.

[23] Nowaczyk MJ (1998). Ethylmalonic and methylsuccinic aciduria in ethylmalonic encephalopathy arise from abnormal isoleucine metabolism. Metabolism.

[24] Ruetz M (2019). Itaconyl-CoA forms a stable biradical in methylmalonyl-CoA mutase and derails its activity and repair. Science.

[25] Eoh H, Rhee KY (2014). Methylcitrate cycle defines the bactericidal essentiality of isocitrate lyase for survival of *Mycobacterium tuberculosis* on fatty acids. Proc Natl Acad Sci U S A.

[26] Zimmermann M (2017). Integration of metabolomics and transcriptomics reveals a complex diet of *mycobacterium tuberculosis* during early macrophage infection. mSystems.

[27] Abrams ME (2020). Oxysterols provide innate immunity to bacterial infection by mobilizing cell surface accessible cholesterol. Nat Microbiol.

[28] Zhou QD (2020). Interferon-mediated reprogramming of membrane cholesterol to evade bacterial toxins. Nat Immunol.

[29] Yang X (2007). Rv1106c from *Mycobacterium tuberculosis* is a 3beta-hydroxysteroid dehydrogenase. Biochemistry.

[30] Yang X (2011). Cholesterol is not an essential source of nutrition for *Mycobacterium tuberculosis* during infection. J Bacteriol.

[31] Brzostek A (2013). ChoD and HsdD can be dispensable for cholesterol degradation in mycobacteria. J Steroid Biochem Mol Biol.

[32] Murphy KC (2018). ORBIT: a new paradigm for genetic engineering of mycobacterial chromosomes. mBio.

[33] van Wyk R (2019). Comprehensive comparative analysis of cholesterol catabolic genes/proteins in mycobacterial species. Int J Mol Sci.

[34] Garton NJ (2008). Cytological and transcript analyses reveal fat and lazy persister-like bacilli in tuberculous sputum. PLoS Med.

[35] Varaksa T (2021). Metabolic fate of human immunoactive sterols in *Mycobacterium tuberculosis*. J Mol Biol.

[36] Köster S (2017). *Mycobacterium tuberculosis* is protected from NADPH oxidase and LC3-associated phagocytosis by the LCP protein CpsA. Proc Natl Acad Sci U S A.

[37] Looney M (2021). Key macrophage responses to infection with *Mycobacterium tuberculosis* are co-regulated by microRNAs and DNA methylation. Front Immunol.

[38] Noubade R (2014). NRROS negatively regulates reactive oxygen species during host defence and autoimmunity. Nature.

[39] McDonald JG (2012). A comprehensive method for extraction and quantitative analysis of sterols and secosteroids from human plasma. J Lipid Res.

[40] Kenny DJ (2020). Cholesterol metabolism by uncultured human gut bacteria influences host cholesterol level. Cell Host Microbe.

[41] Sato Y (2021). Novel bile acid biosynthetic pathways are enriched in the microbiome of centenarians. Nature.

[42] Johnston JB (2010). Functional redundancy of steroid C26-monooxygenase activity in *Mycobacterium tuberculosis* revealed by biochemical and genetic analyses. J Biol Chem.

[43] Knol J (2008). 3-Keto-5alpha-steroid Delta(1)-dehydrogenase from Rhodococcus erythropolis SQ1 and its orthologue in Mycobacterium tuberculosis H37Rv are highly specific enzymes that function in cholesterol catabolism. Biochem J.

[44] Neuvonen M (2014). Enzymatic oxidation of cholesterol: properties and functional effects of cholestenone in cell membranes. PLoS One.

[45] Pucadyil TJ (2005). Membrane cholesterol oxidation inhibits ligand binding function of hippocampal serotonin(1A) receptors. Biochem Biophys Res Commun.

[46] Nguyen DH, Taub DD (2003). Inhibition of chemokine receptor function by membrane cholesterol oxidation. Exp Cell Res.

[47] Elia J (2019). 4-cholesten-3-one decreases breast cancer cell viability and alters membrane raft-localized EGFR expression by reducing lipogenesis and enhancing LXR-dependent cholesterol transporters. Lipids Health Dis.

[48] Xu X, London E (2000). The effect of sterol structure on membrane lipid domains reveals how cholesterol can induce lipid domain formation. Biochemistry.

[49] Kobayashi J (2021). Cholestenone functions as an antibiotic against *Helicobacter pylori* by inhibiting biosynthesis of the cell wall component CGL. Proc Natl Acad Sci U S A.

[50] Marques MA (2015). The essential role of cholesterol metabolism in the intracellular survival of mycobacterium leprae is not coupled to central carbon metabolism and energy production. J Bacteriol.

[51] Rosa TLSA (2021). Reductive power generated by *Mycobacterium leprae* through cholesterol oxidation contributes to lipid and ATP synthesis. Front Cell Infect Microbiol.

[52] Pawełczyk J (2021). Cholesterol-dependent transcriptome remodeling reveals new insight into the contribution of cholesterol to Mycobacterium tuberculosis pathogenesis. Sci Rep.

[53] Costantini C (2020). Tryptophan co-metabolism at the host-pathogen interface. Front Immunol.

[54] Kreit J (2017). Microbial catabolism of sterols: focus on the enzymes that transform the sterol 3β-hydroxy-5-en into 3-keto-4-en. FEMS Microbiol Lett.

[55] Mahmoud HE (2021). Cloning, expression, and in silico structural modeling of cholesterol oxidase of Acinetobacter sp. strain RAMD in E. coli. FEBS Open Bio.

[56] Doukyu N, Nihei S (2015). Cholesterol oxidase with high catalytic activity from Pseudomonas aeruginosa: screening, molecular genetic analysis, expression and characterization. J Biosci Bioeng.

[57] Doukyu N, Aono R (1999). Two moles of O2 consumption and one mole of H2O2 formation during cholesterol peroxidation with cholesterol oxidase from Pseudomonas sp. strain ST-200. Biochem J.

[58] Teng JI, Smith LL (1996). Sterol peroxidation by Pseudomonas fluorescens cholesterol oxidase. Steroids.

[59] Wali H (2019). Cholesterol degradation and production of extracellular cholesterol oxidase from *Bacillus pumilus* W1 and *Serratia marcescens* W8. Biomed Res Int.

[60] Deniz O (2007). Serum total cholesterol, HDL-C and LDL-C concentrations significantly correlate with the radiological extent of disease and the degree of smear positivity in patients with pulmonary tuberculosis. Clin Biochem.

[61] Gebremicael G (2017). Lipid profile in tuberculosis patients with and without human immunodeficiency virus infection. Int J Chronic Dis.

[62] Gagneux S (2012). Host-pathogen coevolution in human tuberculosis. Philos Trans R Soc Lond B Biol Sci.

[63] Stokes WM (1955). The fate of injected cholestenone in the intact rat. J Biol Chem.

[64] Salen G (1984). Transformation of 4-cholesten-3-one and 7 alpha-hydroxy-4-cholesten-3-one into cholestanol and bile acids in cerebrotendinous xanthomatosis. Gastroenterology.

[65] Madenspacher JH (2019). Cholestenoic acid is a prognostic biomarker in acute respiratory distress syndrome. J Allergy Clin Immunol.

[66] Norlin M (2003). On the substrate specificity of human CYP27A1: implications for bile acid and cholestanol formation. J Lipid Res.

[68] Winthrop KL (2020). Incidence and prevalence of nontuberculous mycobacterial lung disease in a large U.S. Managed care health plan, 2008–2015. Ann Am Thorac Soc.

[69] Walzl G (2018). Tuberculosis: advances and challenges in development of new diagnostics and biomarkers. Lancet Infect Dis.

