# Supplementary material for: Macrophage global metabolomics identifies cholestenone as host/pathogen cometabolite present in human Mycobacterium tuberculosis infection
Source: J Clin Invest. 2022 Feb 1;132(3):e152509. doi: 10.1172/JCI152509 (PMC8803325; doi:10.1172/JCI152509)
Supplement: Supplemental data [file jci-132-152509-s177.pdf]

SUPPLEMENTAL MATERIAL

Supplemental Text

Global metabolomics profiling

We performed global metabolite profiling to assess the effect of Mtb infection. Principle component Analysis (PCA) revealed a clear separation based on macrophage infection status at both the early and later time points (**Supplemental Figure 1**). We observed that Mtb infection induced major metabolic perturbations in glucose and cholesterol metabolism, itaconate production, and redox homeostasis. Here we discuss important observations in detail to supplement results shown in the main text.

(i) Nucleotide sugars

Glucose-6-phosphate can be shuttled for the synthesis of nucleotide sugars. We observed increased levels of UDP-glucose and UDP-glucuronate in TB versus Ctrl comparison at both time points (**Supplemental Figure 2**). These metabolites are often used in protein and lipid glycosylation reactions, which in the case of macrophages includes plasma membrane components or proteins destined for export. Nucleotide sugars can also serve as substrates for bacterial glycosyltransferases, which are important for cell wall synthesis.

(ii) Perturbations in redox homeostasis

Macrophage innate immune response involves increased formation of reactive oxygen and nitrogen species (ROS/RNI), which can alter redox balance. Mtb infected macrophages had elevated levels of citrulline levels early after infection (**Supplemental Figure 3A**). This reflects the activity of iNOS, which converts arginine to citrulline and NO. This was accompanied by significant increases in dihydrobiopterin, which represent oxidized forms of tetrahydrobiopterin, a cofactor in NO synthesis (**Supplemental Figure 3B**). Mtb-infected macrophages experienced oxidative stress, accompanied by increases in gamma-glutamyl amino acids (e.g.,

gamma-glutamylglutamine) in infected cells at both time points tested (**Supplemental Figure 3C**). Gamma-glutamyl amino acids are formed when the enzyme gamma-glutamyl transpeptidase transfers the gamma-glutamyl moiety of glutathione to acceptor amino acids. Evidence of oxidative stress experienced by intracellular Mtb was also suggested by marked accumulation of histidine betaine (hercynine) in all infected cells. This compound is a bacterial metabolite that serves as a precursor to ergothioneine, an antioxidant that enables Mtb to withstand oxidative stress of the intracellular milieu (**Supplemental Figure 3D**)

(iii) Other notable changes

We observed elevated 1-methyl-5-imidazoleacetate in all infected samples compared to controls (**Supplemental Figure 4A**). The expression of histidine decarboxylase (HDC) can be induced in macrophages by inflammatory stimuli resulting in formation of histamine. Interestingly, elevated serotonin was also observed in TB versus Ctrl 24 hpi (**Supplemental Figure 4B**).

Supplemental Methods

Global metabolomics profiling

Metabolite Quantification and Data Normalization: Peaks were quantified using area-under-the-curve. For studies spanning multiple days, a data normalization step was performed to correct variation resulting from instrument inter-day tuning differences. Essentially, each compound was corrected in run-day blocks by registering the medians to equal one (1.00) and normalizing each data point proportionately (termed the "block correction"). For studies that did not require more than one day of analysis, no normalization is necessary, other than for purposes of data visualization. In certain instances, biochemical data may have been normalized to an additional factor (e.g., cell counts, total protein as determined by Bradford assay, osmolality, etc.) to account for differences in metabolite levels due to differences in the amount of material present in each sample.

Data analysis: The presented dataset comprises a total of 507 compounds of known identity (**Supplemental Table 1**). For each biochemical, the "OrigScale" values are the area under the curve values for each peak. Each biochemical in OrigScale was rescaled to set the median equal to 1, which was referred to as ScaledImpData or scaled intensity in the box plots. Log transformation and imputation of missing values, if any, was done with the minimum observed value for each compound, ANOVA contrasts were used to identify biochemicals that differed significantly between experimental groups. A summary of the numbers of biochemicals that achieved statistical significance ($p \leq 0.05$), as well as those approaching significance ($0.05 < p < 0.10$), is shown indicated in **Supplemental Table 1**. Analysis by ANOVA identified biochemicals exhibiting significant interaction and main effects for experimental parameters of infection and timepoint. An estimate of the false discovery rate (q-value) was calculated to take

into account the multiple comparisons that normally occur in metabolomic-based studies (**Supplemental Table 1**).

Molecular biology

Plasmid extractions and DNA purifications were done using the QIAPrep spin Miniprep Kit (Qiagen) and QIA quick PCR purification kit (Qiagen) respectively, according to the manufacturer's instructions. Polymerase chain reactions (PCR) were carried out using the high fidelity Phusion DNA polymerase (New England BioLabs). When mentioned, one-step assembly of linear vectors and inserts were carried out using the NEBuilder HiFi DNA Assembly kit (New England BioLabs). Restriction enzymes were used according the manufacturer's instructions. Plasmids and primers used in this study can be found in **Supplemental Table 5** and **6**. The pKM464 plasmid was a gift from Kenan Murphy (Addgene plasmid # 108322). The pKM461 plasmid was a gift from Kenan Murphy (Addgene plasmid # 108320). Extraction of genomic DNA was done by cetyltrimethylammonium bromide-lysozyme lysis, followed by chloroform-isoamyl alcohol extraction and isopropanol precipitation, as previously described (1).

Construction of mutants and complemented strains

Rv1106c/3β-*hsd* and *Rv3409c/choD* were deleted in the H37Rv strain using plasmids pKM461 and pKM464 (**Supplemental Table 5**), and HC291 or HC292 primers (**Supplemental Table 6**), respectively, using the ORBIT method described in (2). The HC291 and HC292 primers were designed according to the rules described in (2), with homology regions annealing upstream and downstream of the target gene flanking an *attP* site. For the construction of the Δ*hsd* mutant, H37Rv containing pKM461 was grown in liquid media and incubated with

anhydrous tetracycline (500 ng/mL) for 16-20 hours before co-electroporation of HC292 and pKM464. Hygromycin-resistant colonies were screened for integration of pKM464 in the Rv1106c open reading frame by PCR using primers HC338/HC343 (**Supplemental Table 6**), leading to amplification of DNA fragments of 3500 bp in the mutant and 1500 bp in the parental strain. For deletion of *choD* (Rv3409c), we electroporated HC291 and pKM464 in H37Rv containing pKM461. Insertion of pKM464 in *Rv3409c* was verified by PCR using oligos HC336 and HC342 (**Supplemental Table 6**), which amplify fragments of 3440 bp in the mutant and 2090 bp in the parental strain.

Due to the complexity of the genetic region of Rv1106c, complementation of the Δ*hsd* mutant was designed by cloning the entire operon on an integrative plasmid, starting from the intergenic region upstream of Rv1109c, followed by Rv1108c (*xseA*), Rv1107c (*xseB*) and Rv1106c. Primers HC354/HC357 were used to amplify a 3500 bp DNA fragment from pKP617. Using primers HC353/HC356, the 3500 bp genetic region of from Rv1109c-Rv1106c was amplified from H37Rv genomic DNA and subsequently assembled with the linearized fragment of pKP617 using NEBuilder HiFi DNA Assembly, leading to plasmid pCH89. Correct assembly of pCH89 was confirmed by enzymatic restriction, and the full insert was sequenced to confirm no mutations were introduced. The pCH89 complementation plasmid was then electroporated in H37Rv (pKM461) and Δ*hsd* (pKM461, pKM464).

Cholestenone concentration determination

For data reported in Figure 3B and Figure 4G, 4-cholesten-3-one was quantified on a Q Exactive mass spectrometer. Data processing was conducted with Xcalibur 4.2 and the area ratio of 4-cholesten-3-one to the internal standard (d_5-4-cholesten-3-one) was used to

determine the 4-cholesten-3-one concentration. All other cholestenone measurements (Figures 3B, 3C, 3E; Figures 4C, 4E, 4F; Figures 5-7) were performed on a 4000QTRAP mass spectrometer. In these cases, the 4-cholesten-3-one concentration was calculated by converting the area ratio to concentration with a standard curve (**Supplemental Figure 6A)**. The standard curve was obtained by analysis of samples containing 4-cholesten-3-one (0.01 ng/mL to 1000 ng/mL) in 80% methanol with 0.2 μg/mL d_5-4-cholesten-3-one. Samples were analyzed in technical duplicates with a 4000QTRAP mass spectrometer. The standard curve was established using the software Analyst 1.6.3 (AB Sciex, Vaughan, Canada). For plasma and sputum samples, the concentration was subsequently corrected to take into account the sample dilution introduced by sample processing (5-fold and 10-fold, respectively). Throughout the manuscript, metabolite abundance is reported from unprocessed samples (plasma or non-liquefied sputum). The standard curve was >96% accurate for values above 2.5 ng/mL for plasma and 5 ng/mL for sputum. 29 sputum samples (25 TB negative; 4 TB positive) fell below this accuracy threshold, so were adjusted to 5 ng/ml for further statistical analysis. This conservative adjustment means that differences between groups is an underestimate of the true difference.

Cholesterol estimation and cell viability assay

Total cholesterol, total protein, and cell viability were measured using Amplex Red Cholesterol Assay kit (Invitrogen cat. No. A12216), Pierce BCA Protein Assay Kit (Thermofisher Scientific cat. No. 23252), and CellTiter Glo Luminescent Cell Viability Assay kit (Promega cat. No. G9241), respectively.

Statistical analysis

Violin plots and ROC plots were generated using RStudio version 1.3.1073 (3), with the following packages: ggplot2 (4), pROC (5), ggpubr (6), and egg (7).

Supplemental References

1. Larsen MH, Biermann K, Tandberg S, Hsu T, and Jacobs WR. Genetic Manipulation of *Mycobacterium tuberculosis*. *Curr Protoc Microbiol.* 2007;Chapter 10:Unit 10A.2.
2. Murphy KC, Nelson SJ, Nambi S, Papavinasasundaram K, Baer CE, and Sassetti CM. ORBIT: a New Paradigm for Genetic Engineering of Mycobacterial Chromosomes. *mBio.* 2018;9(6).
3. R StudioTeam. RStudio: Integrated Development Environment for R. RStudio, PBC, Boston, MA (2020). *URL* ***http://www.rstudio.com/****.*
4. Wickham H (2016). ggplot2: Elegant Graphics for Data Analysis. Springer-Verlag New York. ISBN 978-3-319-24277-4, https://ggplot2.tidyverse.org.
5. Robin X, N. Turck N, Hainard A, Tiberti N, Lisacek F, Sanchez JC and Müller M. pROC: an open-source package for R and S+ to analyze and compare ROC curves. BMC Bioinformatics, 12, p. 77. (2011)
6. Kassambara A. ggpubr: 'ggplot2' Based Publication Ready Plots. (2020). R package version 0.4.0. https://CRAN.R-project.org/package=ggpubr
7. Auguie B. egg: Extensions for 'ggplot2': Custom Geom, Custom Themes, Plot Alignment, Labelled Panels, Symmetric Scales, and Fixed Panel Size. (2019) R package version 0.4.5. https://CRAN.R-project.org/package=egg
8. Köster S, Upadhyay S, Chandra P, Papavinasasundaram K, Yang G, Hassan A, et al. *Mycobacterium tuberculosis* is protected from NADPH oxidase and LC3-associated

phagocytosis by the LCP protein CpsA. *Proc Natl Acad Sci U S A.* 2017;114(41):E8711-E20.

Supplemental Figures

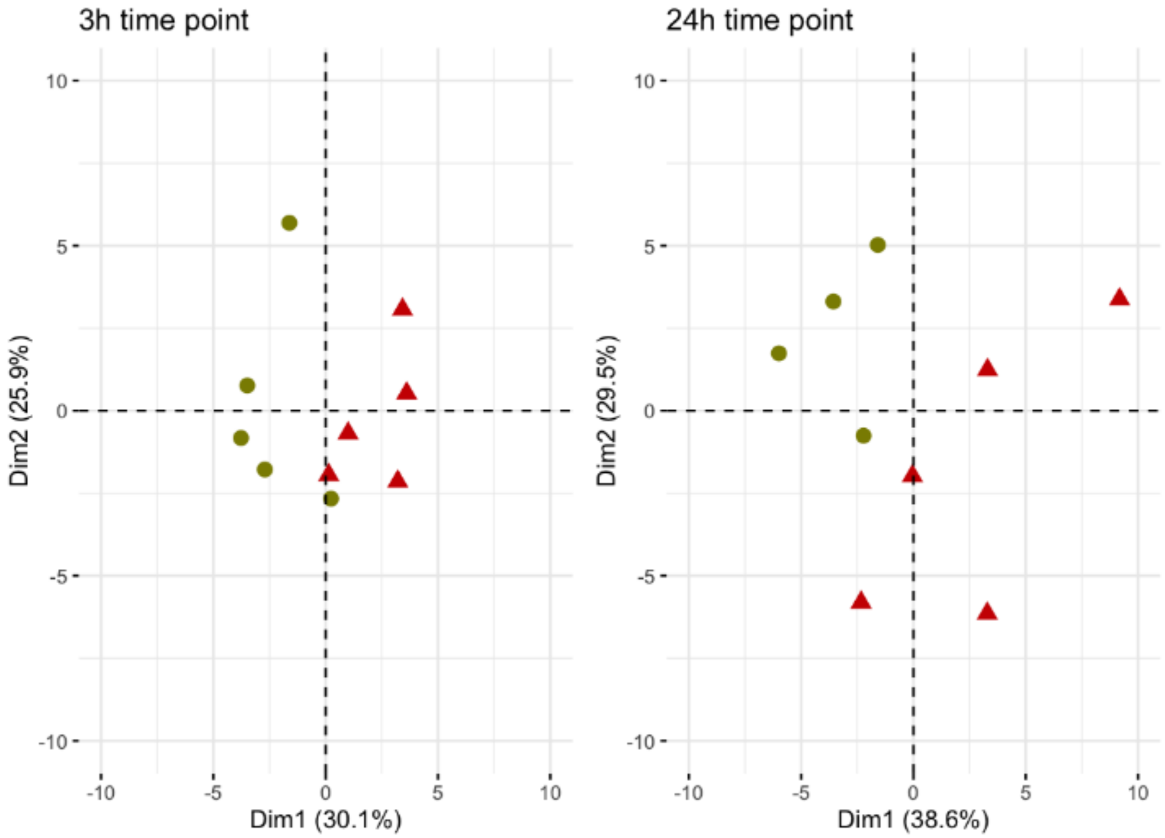

Supplemental Figure 1. Principal component analysis (PCA) of global metabolomics data showed a clear separation between uninfected (green circles) and Mtb-infected (red triangles) macrophages at both time points. Each point in the plot represents a replicate for a sample group. For each group, we used 5 replicates to generate results. One of five replicates for the uninfected 24h group was considered an outlier, and was excluded from subsequent analyses.

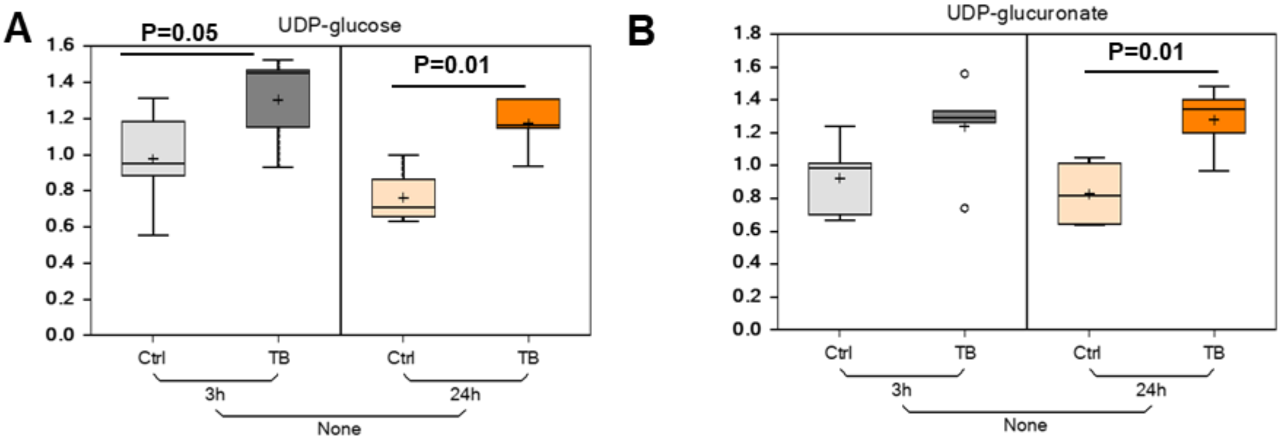

Supplemental Figure 2. Infection-induced increases in nucleotide sugars. The box plots show levels of **(A)** UDP-glucose and **(B)** UDP-glucuronate in uninfected (Ctrl) and Mtb-infected (TB) macrophages 3 and 24 hpi. Metabolite levels are shown in box plots as scaled intensity. For each metabolite, the raw area counts were rescaled to set the median equal to 1. Plots show median as a line and mean as '+'. Open circles show extreme data points in the group. Data analysis was done by ANOVA.

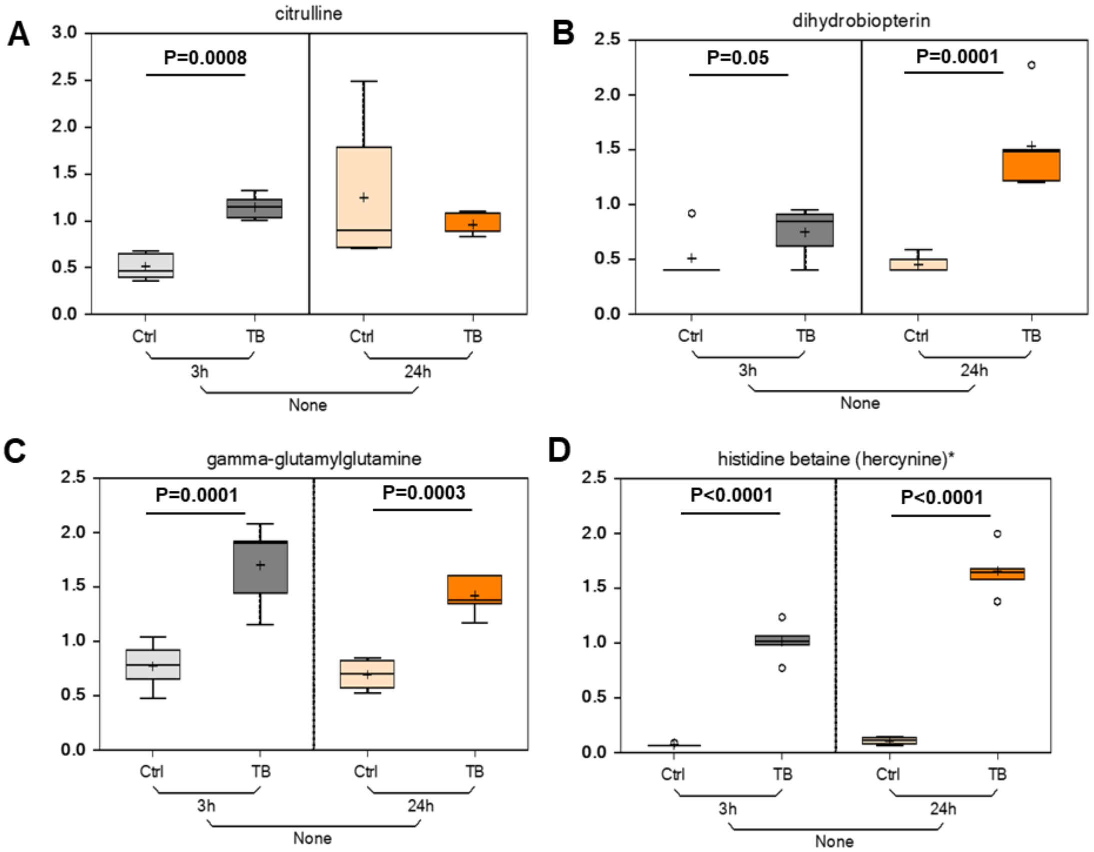

Supplemental Figure 3. Infection-induced perturbations in redox homeostasis. Box plots showing scaled intensity for **(A)** citrulline **(B)** dihydrobiopterin **(C)** gamma-glutamylglutamine and **(D)** histidine betaine (hercynine) in uninfected (Ctrl) and Mtb-infected (TB) macrophages 3 and 24 hpi. Box plots show median as a line and mean as '+'. Open circles show extreme data points. Analysis was done by ANOVA.

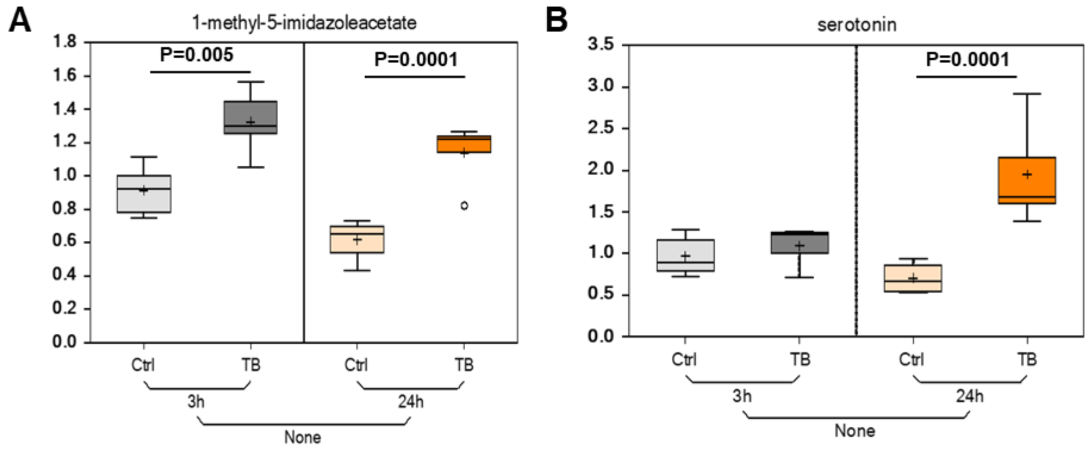

Supplemental Figure 4. Other changes induced by Mtb infection of macrophages. We observed significant elevations in levels of **(A)** 1-methyl-5-imidazoleacetate and **(B)** serotonin in TB versus Ctrl 24hpi. Box plots show indicated metabolite levels as scaled intensity, median as a line, and mean as '+'. Open circles show extreme data points. Analysis was done by ANOVA. Ctrl=uninfected, TB=Mtb-infected.

A

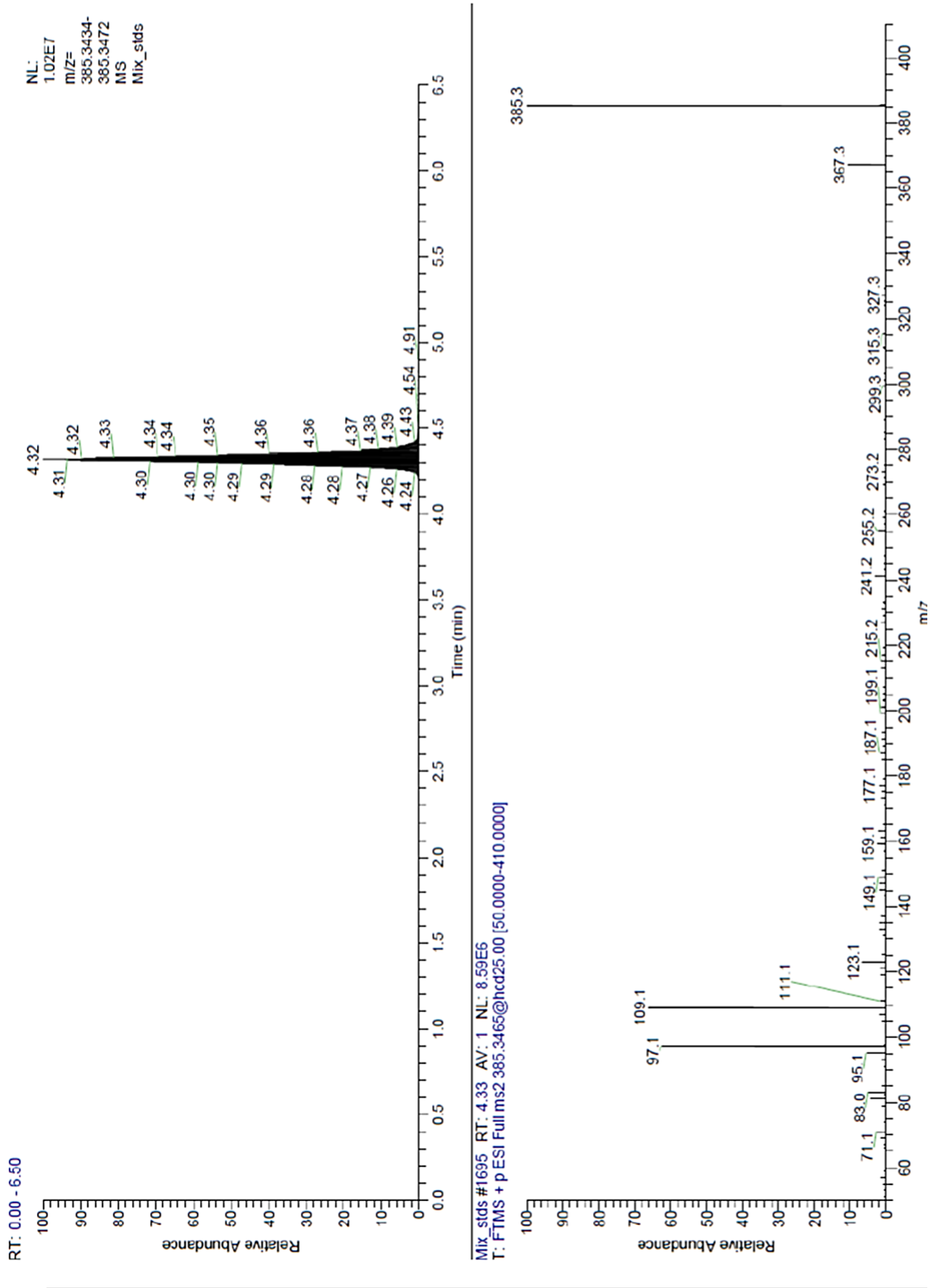

RT: 0.00 - 6.50
NL: 1.02E7 m/z= 385.3434-385.3472 MS Mix_stds
Relative Abundance
Time (min)
Mix_stds #1695 RT: 4.33 AV: 1 NL: 8.59E6
T: FTMS + p ESI Full ms2 385.3465@hcd25.00 [50.0000-410.0000]
m/z

B

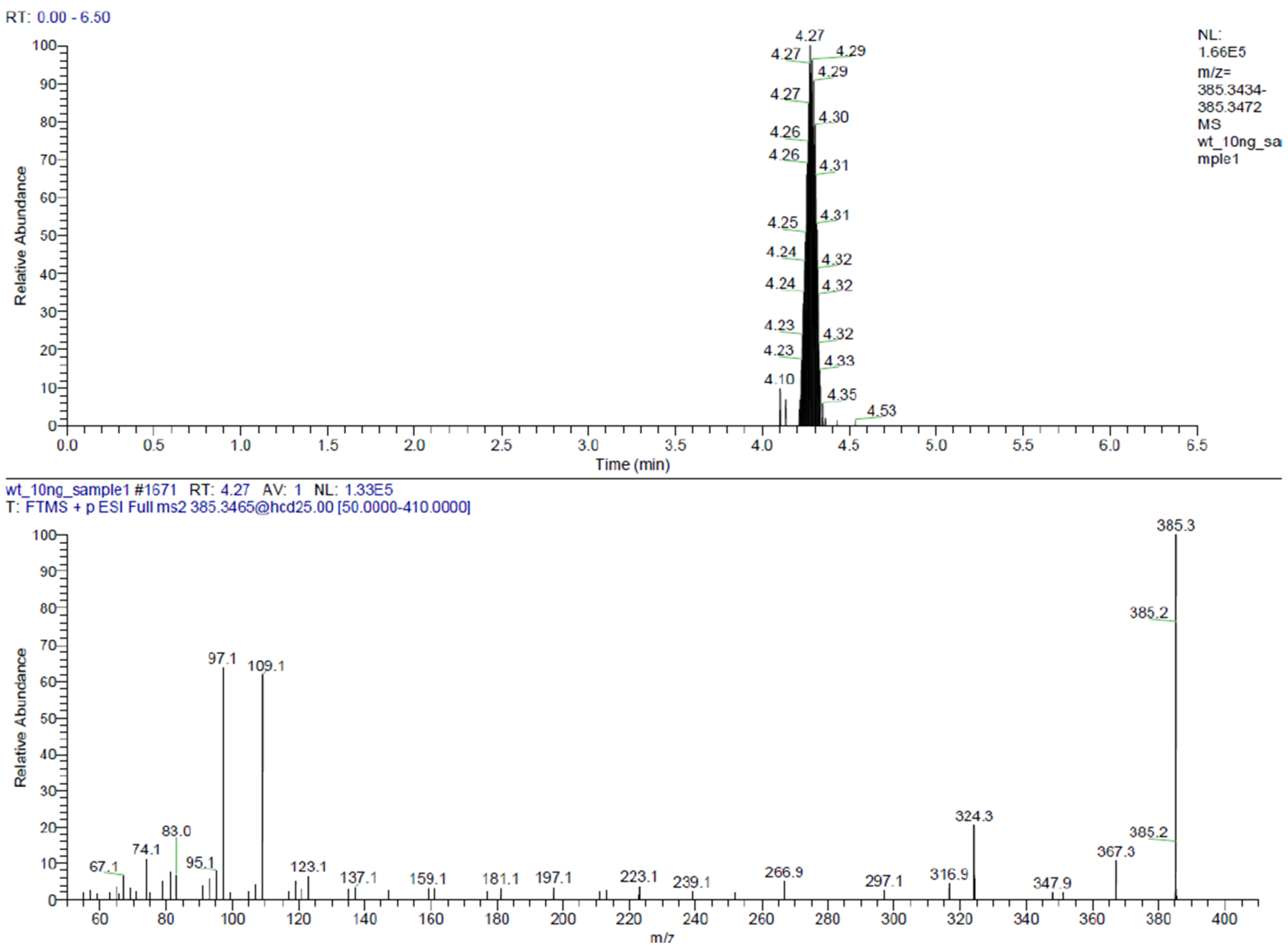

RT: 0.00 - 6.50
NL: 1.66E5 m/z= 385.3434-385.3472 MS wt_10ng_sample1
4.27
4.29
4.30
4.31
4.32
4.33
4.35
4.53
4.26
4.25
4.24
4.23
4.10
Relative Abundance
Time (min)
wt_10ng_sample1 #1671 RT: 4.27 AV: 1 NL: 1.33E5
T: FTMS + p ESI Full ms2 385.3465@hcd25.00 [50.0000-410.0000]
385.3
385.2
97.1
109.1
324.3
367.3
83.0
74.1
95.1
67.1
123.1
137.1
159.1
181.1
197.1
223.1
239.1
266.9
297.1
316.9
347.9
m/z

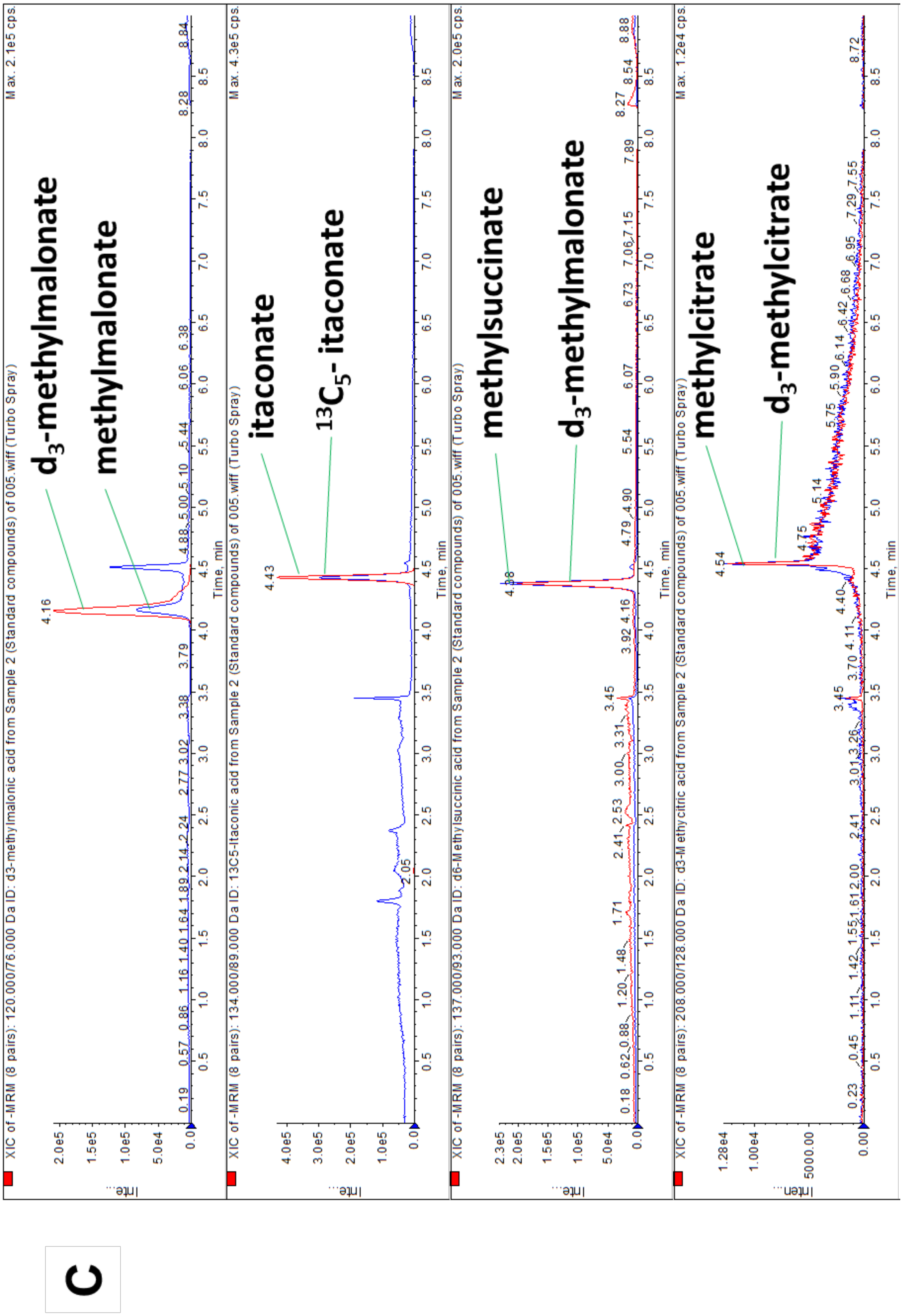
C
d_3-methylmalonate
methylmalonate
itaconate
$^{13}C_5$- itaconate
methylsuccinate
d_3-methylmalonate
methylcitrate
d_3-methylcitrate

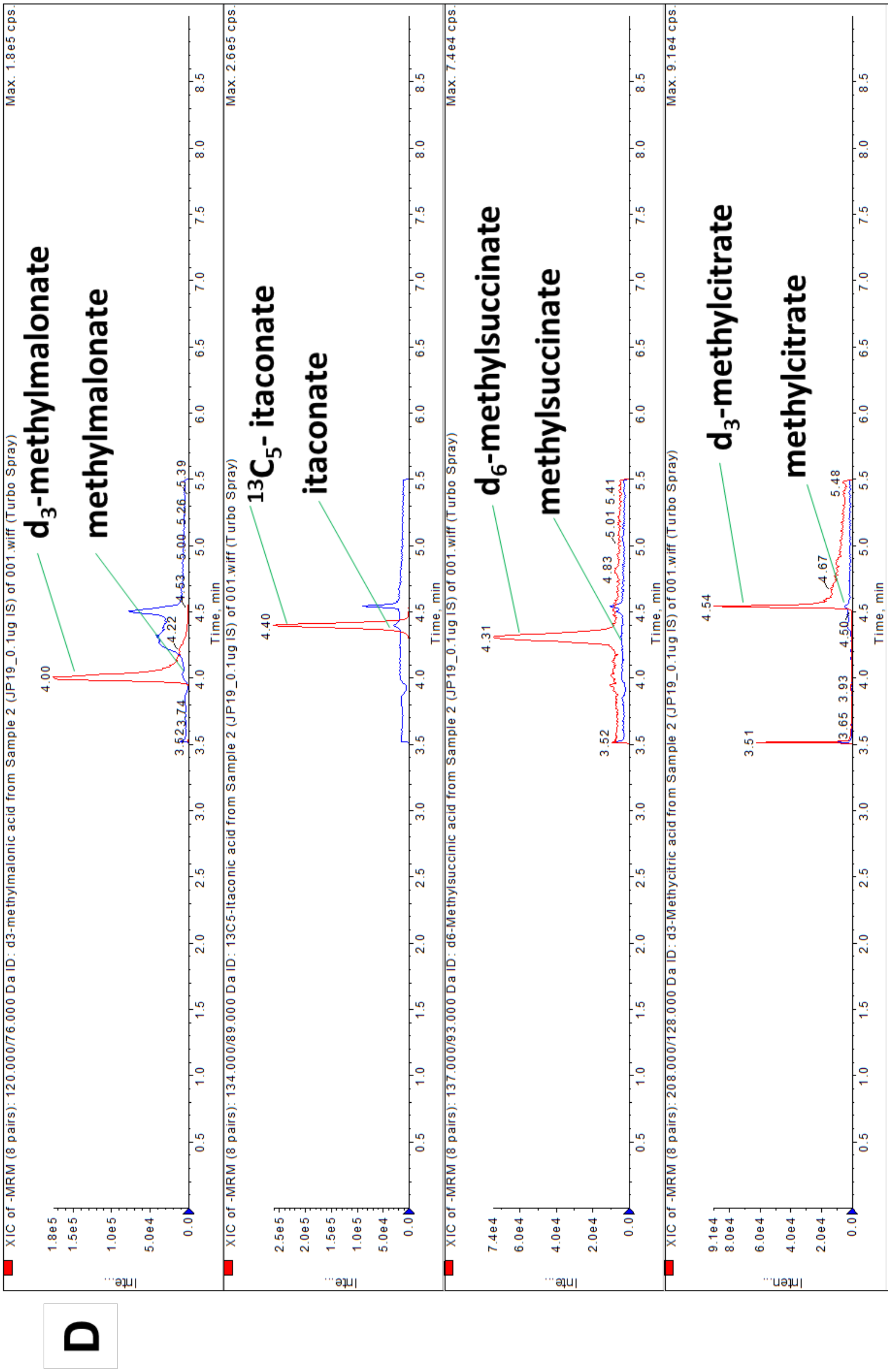
D
XIC of -MRM (8 pairs): 120.000/76.000 Da ID: d3-methylmalonic acid from Sample 2 (JP19_0.1ug IS) of 001.wiff (Turbo Spray)
Max. 1.8e5 cps.
d_3-methylmalonate
methylmalonate
XIC of -MRM (8 pairs): 134.000/89.000 Da ID: 13C5-Itaconic acid from Sample 2 (JP19_0.1ug IS) of 001.wiff (Turbo Spray)
Max. 2.6e5 cps.
$^{13}C_5$- itaconate
itaconate
XIC of -MRM (8 pairs): 137.000/93.000 Da ID: d6-Methylsuccinic acid from Sample 2 (JP19_0.1ug IS) of 001.wiff (Turbo Spray)
Max. 7.4e4 cps.
d_6-methylsuccinate
methylsuccinate
XIC of -MRM (8 pairs): 208.000/128.000 Da ID: d3-Methycitric acid from Sample 2 (JP19_0.1ug IS) of 001.wiff (Turbo Spray)
Max. 9.1e4 cps.
d_3-methylcitrate
methylcitrate
Time, min

Supplemental Figure 5. Mass spectrometry validation of reported metabolites. (**A**) Chromatogram and mass spectrometry spectra of the 4-cholesten-3-one reference compound. (**B**) Chromatogram and mass spectrometry spectra of 4-cholesten-3-one in macrophage extracts. (**C**) Combined chromatograms for the methylmalonate, itaconate, methylsuccinate, methylcitrate standard compounds (shown in blue) and their respective internal standards (shown in red). (**D**) Combined chromatograms for the methylmalonate, itaconate, methylsuccinate, and methylcitrate metabolites identified from macrophage extracts (shown in blue) with their respective internal standards (shown in red).

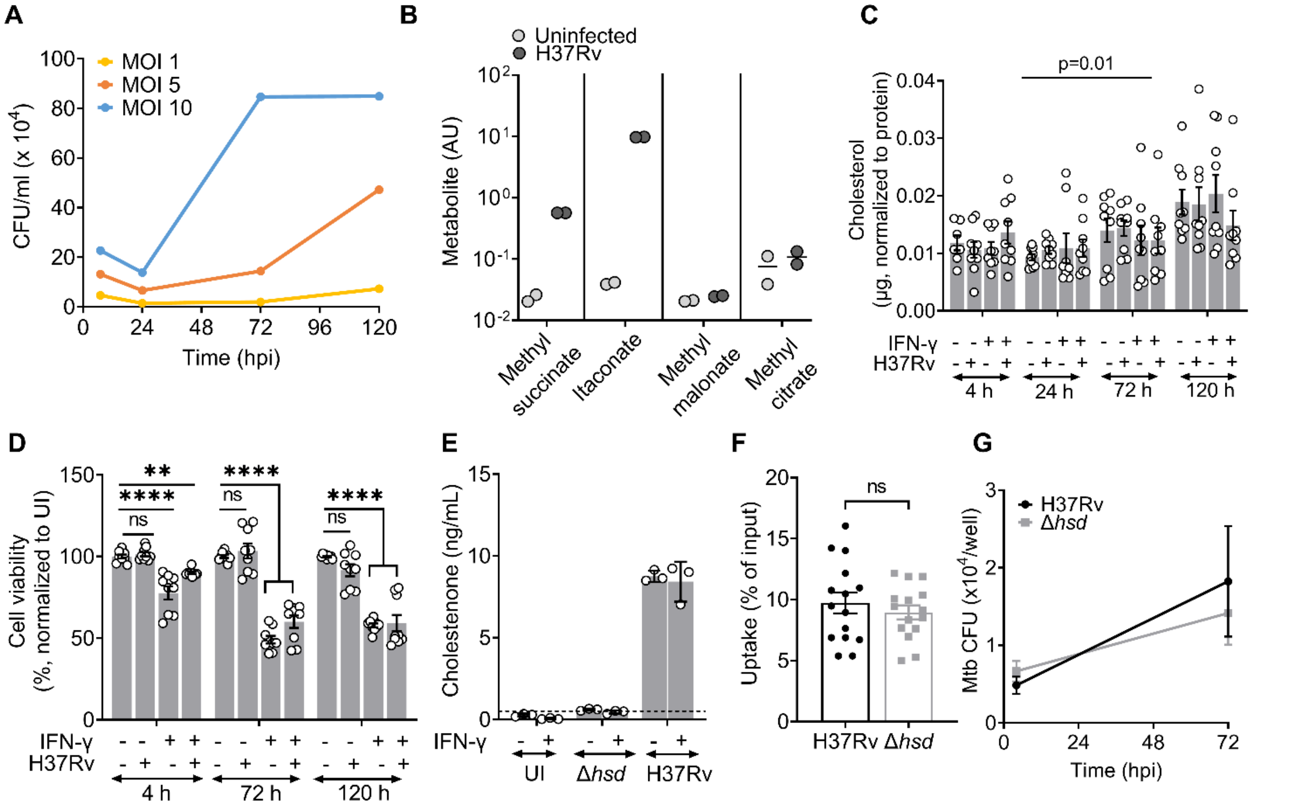

Supplemental Figure 6. ***M. tuberculosis*** **infection of BMDM: colony forming units (CFU), metabolite levels, and cell viability.** (**A**) Mtb CFU were quantified in BMDMs infected at MOI 1, 5, and 10 at indicated time points. Values are average of at least 3 replicates from one experiment. (**B**) Levels of indicated metabolites were compared in BMDMs that were uninfected or infected with H37Rv at MOI 10, 72 hpi. Data are from one experiment with technical duplicates. (**C**) Cholesterol levels and (**D**) cell viability in IFNγ-activated and naïve BMDMs that were uninfected or Mtb-infected at MOI 10 at the indicated time points. The cholesterol concentrations (µg/mL) were normalized to protein concentration (µg/mL) in the sample. Cholesterol values were not significantly different between samples except between uninfected 24h versus uninfected 120h ($p=0.01$). (**C**, **D**) Plots show mean +/- s.e.m. from three independent experiments. **$p=0.007$, ****$p<0.0001$ calculated using one-way ANOVA with Tukey's multiple comparisons test in (**C**) and Dunnett's multiple comparisons test in (**D**). **(E)** Cholestenone was measured in IFN-γ-activated and naïve BMDMs that were uninfected (UI) or infected with wild-type or Δ*hsd* mutant Mtb at MOI 10 for 72 h. Plot shows mean +/- s.d. of one experiment (N=3). The dotted line shows the limit of detection accuracy as determined by the standard curve (details in Supplemental Materials). (**F-G**) Bacterial CFU were quantified in PMA-differentiated THP-1 macrophages infected with wild-type or Δ*hsd* mutant Mtb at MOI 5 to assess (**F**) bacterial uptake and (**G**) intracellular bacterial growth. Plot shows (**F**) mean +/- s.e.m. from three independent experiments measured 4 hours after bacterial addition and (**G**) mean +/- s.d. of five replicates of one experiment, representative of three independent experiments. For all macrophage experiments, one million cells were infected, and the samples were extracted in 500 µL of 80 % methanol solution at indicated timepoints.

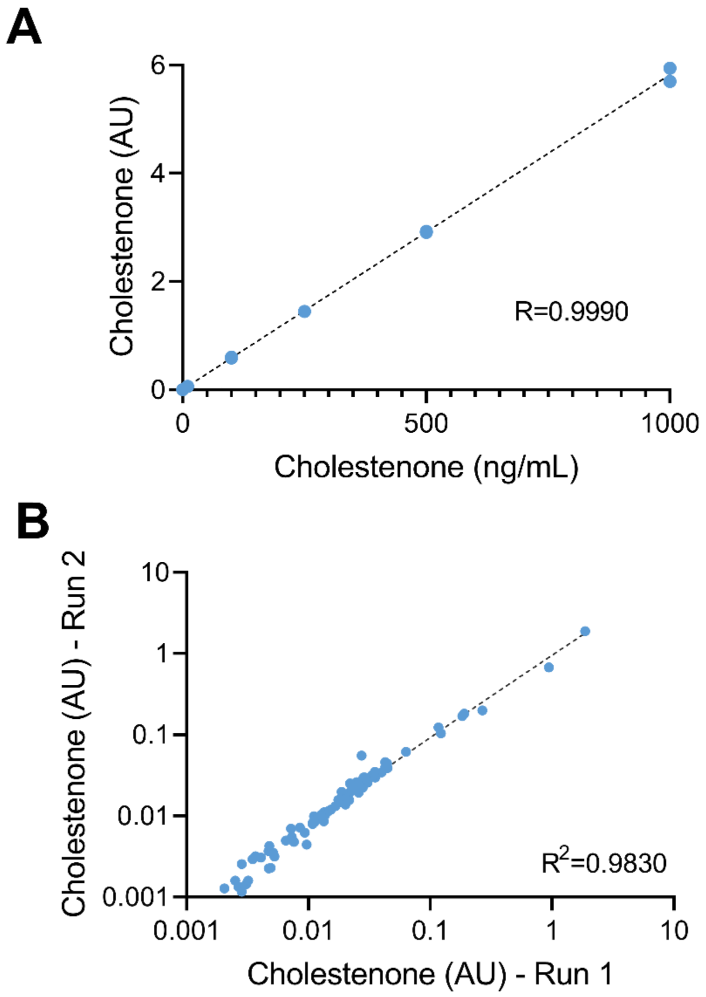

Supplemental Figure 7. Standard curve for cholestenone quantification and correlation between replicates. (**A**) The known concentration of cholestenone (ng/ml) was compared to area ratios calculated by mass spectrometry to establish a standard curve; the linear regression equation is as follows: y = 0.00593 x + 0.00163 (r = 0.990). (**B**) Cholestenone quantification is plotted on each axis for the results of two independent extractions (run 1 and run 2). R^2 obtained by linear regression. AU= arbitrary units, calculated from area ratio of metabolite peak versus internal standard.

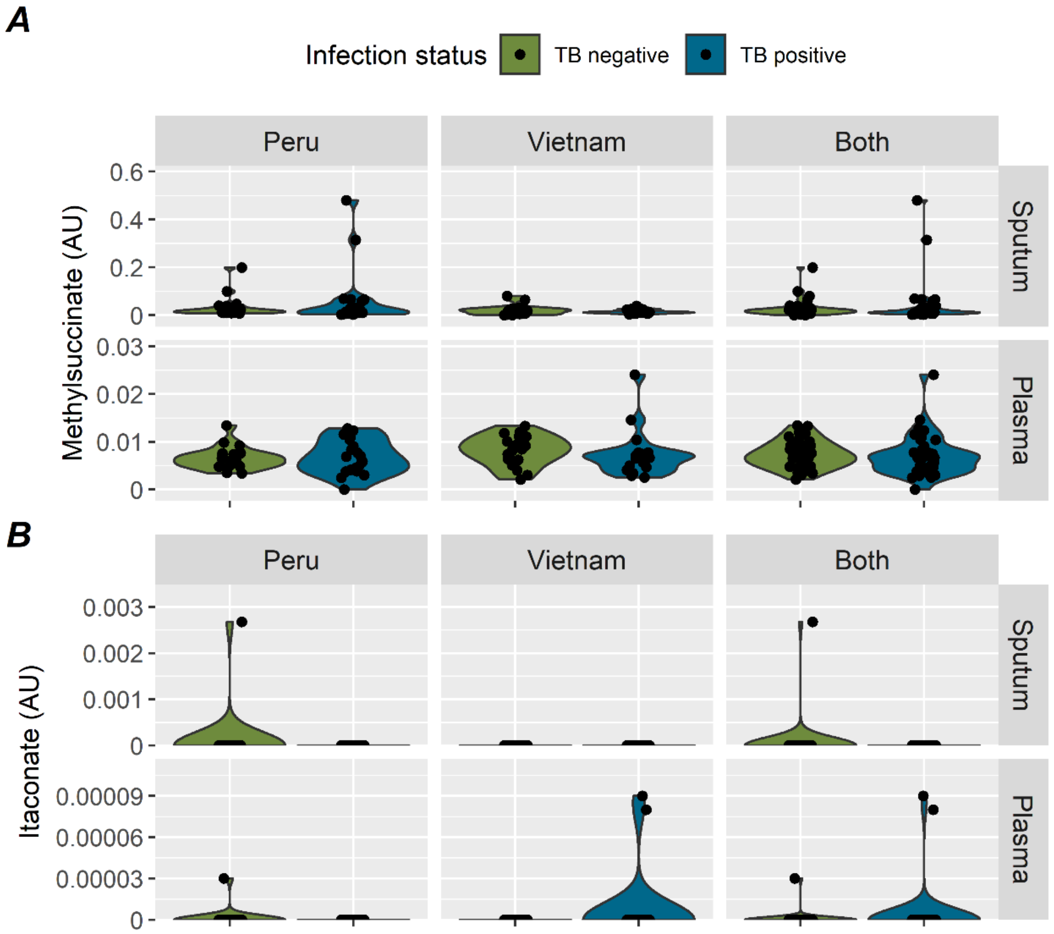

Supplemental Figure 8. Methylsuccinate and itaconate levels do not correlate with Mtb infection status. (**A**) Methylsuccinate and (**B**) itaconate levels in sputum and plasma samples plotted based on infection status. Statistical analyses were done using the Mann-Whitney test for sputum samples and Student's t-tests for plasma samples. AU= arbitrary units, calculated from area ratio of metabolite peak versus internal standard.

.

Supplemental Tables

Supplemental Table 1.

Included as a separate excel file.

Supplemental Table 2. Summary statistics for clinical variables by country.

Variable		Peru (N = 40)	Vietnam (N = 40)	P value*
Infection status	TB Negative	20/40 (50%)	20/40 (50%)	>0.999
	TB Positive	20/40 (50%)	20/40 (50%)	
Sex	Female	15/40 (38%)	12/40 (30%)	0.478
	Male	25/40 (62%)	28/40 (70%)	
Age, mean (SD); range		32 (11.2) 19-56	49 (14.8) 20-77	<0.001
History of BCG vaccination		38/39 (97%)	22/32 (69%)	0.002
History of active TB		1/28 (4%)	8/40 (20%)	0.071
Normal chest x-ray		15/36 (42%)	0/40 (0%)	<.001
Chest pain		31/40 (78%)	20/40 (50%)	0.011
Dyspnea		25/40 (63%)	22/40 (55%)	0.496
Fever		17/40 (43%)	25/40 (63%)	0.073
Hemoptysis		11/40 (28%)	9/40 (23%)	0.606
Malaise		39/40 (98%)	30/40 (75%)	0.007
Night sweats		16/40 (40%)	15/40 (38%)	0.818
Cough		40/40 (100%)	40/40 (100%)	>0.999
Recent weight loss (without diet)		22/40 (55%)	17/40 (43%)	0.263

Results reported as n (%) or mean (range). * P values obtained using a chi-square test, Fisher's exact test, or t-test, as appropriate.

Supplemental Table 3. Associations between metabolite abundance and clinical parameters for TB positive patients

Outcome: Cholestenone in sputum (ng/mL)								
Variable	Group	N	Mean	Std Dev	Median	Q1	Q3	P value†
Sex	FEMALE	11	331.3	949.3	24.6	5.0	46.3	0.355
	MALE	29	111.8	221.8	32.3	15.0	83.0	
Chest Pain	NO	16	336.0	812.2	29.3	19.4	208.3	0.288
	YES	24	62.9	84.1	30.2	7.9	74.8	
Dyspnea	NO	18	57.3	90.2	24.4	15.0	66.3	0.828
	YES	22	266.1	697.0	31.6	9.3	203.7	
Fever	NO	16	154.4	285.0	39.7	19.3	143.3	0.219
	YES	24	184.0	644.3	26.1	7.9	60.9	
Hemoptysis	NO	27	214.4	634.3	30.9	15.0	82.7	0.544
	YES	13	84.5	117.7	18.5	9.3	90.6	
Night Sweats	NO	19	123.8	265.6	28.1	13.9	82.7	0.828
	YES	21	215.9	686.3	30.9	10.6	90.6	
Recent Weight Loss	NO	14	158.7	304.3	37.2	15.0	83.0	0.514
	YES	26	179.4	618.6	27.5	13.5	67.0	
Sputum grade	2+	16	18.7	14.0	14.4	6.0	29.3	0.001
	3+	24	274.5	664.1	66.7	23.8	242.7	
Outcome: Cholesterol in sputum (µg/mL)								
Variable	Group	N	Mean	Std Dev	Median	Q1	Q3	P value‡
Sex	FEMALE	11	268.6	112.9	303.0	160.1	351.6	0.080
	MALE	29	196.5	113.3	164.2	99.0	308.7	
Chest Pain	NO	16	187.4	128.4	144.8	79.0	332.5	0.204
	YES	24	235.6	106.1	279.1	154.4	319.3	
Dyspnea	NO	18	206.4	117.8	187.4	121.7	313.8	0.631
	YES	22	224.5	117.3	266.9	114.2	339.2	
Fever	NO	16	197.0	122.0	162.1	85.6	325.2	0.398
	YES	24	229.2	113.3	266.9	128.7	327.1	
Hemoptysis	NO	27	217.9	117.6	227.9	114.2	324.7	0.902
	YES	13	213.0	118.6	214.8	142.8	339.2	
Night Sweats	NO	19	206.1	116.3	167.7	99.0	324.7	0.605
	YES	21	225.5	118.5	262.6	121.7	340.3	
Recent Weight Loss	NO	14	182.2	120.3	148.7	72.2	297.1	0.176
	YES	26	234.7	112.3	266.9	142.8	339.2	
Sputum grade	2+	16	228.4	110.2	249.5	128.7	332.5	0.599

	3+	24	208.3	122.0	187.4	85.6	316.7	

Outcome: Cholestenone in plasma (ng/mL)								
Variable	Group	N	Mean	Std Dev	Median	Q1	Q3	P value‡
Sex	FEMALE	11	16.9	9.3	12.3	10.4	20.6	0.690
	MALE	29	15.6	9.3	12.2	7.9	23.6	
Chest Pain	NO	16	15.9	10.2	12.3	8.0	22.6	0.976
	YES	24	16.0	8.7	12.9	8.3	23.6	
Dyspnea	NO	18	15.2	10.1	10.4	7.5	25.5	0.659
	YES	22	16.5	8.6	15.0	8.8	22.4	
Fever	NO	16	15.9	9.6	12.6	8.0	23.6	0.988
	YES	24	15.9	9.1	12.1	8.6	23.0	
Hemoptysis	NO	27	16.9	10.2	12.2	8.1	26.3	0.353
	YES	13	13.9	6.5	13.9	7.1	19.7	
Night Sweats	NO	19	14.4	9.0	11.6	7.9	19.7	0.336
	YES	21	17.3	9.4	16.1	8.7	23.6	
Recent Weight Loss	NO	14	13.7	7.8	11.9	8.1	13.9	0.260
	YES	26	17.1	9.8	16.2	8.5	23.6	
Sputum grade	2+	16	15.9	9.5	12.0	8.3	22.6	0.980
	3+	24	16.0	9.2	12.6	8.1	23.6	

Outcome: Cholesterol in plasma (µg/mL)								
Variable	Group	N	Mean	Std Dev	Median	Q1	Q3	P value‡
Sex	FEMALE	11	831.5	239.9	834.5	681.0	897.6	0.767
	MALE	29	860.4	283.7	822.7	697.9	1029.8	
Chest Pain	NO	16	836.6	203.6	847.0	731.4	939.3	0.766
	YES	24	863.0	309.8	816.7	677.1	1021.4	
Dyspnea	NO	18	824.8	170.3	818.7	748.0	888.3	0.542
	YES	22	875.0	332.3	881.6	673.2	1052.6	
Fever	NO	16	845.8	221.8	818.7	731.4	981.3	0.901
	YES	24	856.9	302.0	850.2	677.1	979.5	
Hemoptysis	NO	27	915.0	244.3	871.8	783.9	1029.8	0.033
	YES	13	722.5	282.8	681.0	507.6	810.7	
Night Sweats	NO	19	850.9	284.4	814.8	698.8	932.8	0.973
	YES	21	853.8	262.7	865.8	681.0	1013.0	
Recent Weight Loss	NO	14	919.9	334.7	850.2	685.2	1084.8	0.250
	YES	26	816.1	226.4	818.7	697.9	945.9	
Sputum grade	2+	16	840.7	290.5	806.9	689.9	939.3	0.826
	3+	24	860.2	261.0	825.4	691.6	1047.2	

Clinical parameters were assessed for statistical association with metabolite levels in samples from TB positive subjects of both countries (n=40). Metabolite abundance is reported in unprocessed samples (plasma or non-liquefied sputum). † Mann-Whitney test; ‡ t-test

Supplemental Table 4. Cholesterol and cholestenone concentrations in clinical samples.

Cholesterol (µg/mL)							
**Sample type**	**Country**	**Infection status**	**n**	**Range**	**Mean**	**SD**	**Median**
Plasma	Peru	TB -	20	751.9 - 1577.7	1027.6	232.0	945.6
		TB +	20	397.1 - 1368.3	791.8	270.9	740.7
	Vietnam	TB -	20	883.1 - 1618.5	1190.5	241.1	1127.6
		TB +	20	440.9 - 1701.0	913.1	261.1	881.7
Sputum (non-liquefied)	Peru	TB -	20	127.0 - 386.6	283.4	94.0	298.0
		TB +	20	32.3 - 379.9	283.7	109.6	325.2
	Vietnam	TB -	20	6.0 - 386.0	177.0	113.2	163.3
		TB +	20	33.8 - 297.1	149.0	79.0	144.8
**Cholestenone (ng/mL)**							
**Sample type**	**Country**	**Infection status**	**n**	**Range**	**Mean**	**SD**	**Median**
Plasma	Peru	TB -	20	5.1 - 31.7	13.4	7.6	11.8
		TB +	20	7.0 - 37.4	22.1	7.9	23.0
	Vietnam	TB -	20	5.5 - 20.3	11.2	4.4	10.4
		TB +	20	6.1 - 32.1	9.8	5.6	8.3
Sputum (non-liquefied)	Peru	TB -	20	< 5.0*[1] - 172.1	19.1	37.2	6.8
		TB +	20	< 5.0*[2] - 3183.5	290.7	728.2	27.1
	Vietnam	TB -	20	< 5.0*[3] - 14.2	6.0	2.4	5.0
		TB +	20	< 5.0*[4] - 399.7	53.6	84.9	29.5

Cholestenone concentration was determined as described in Supplemental Methods. 29 sputum samples have cholestenone values below 5 ng/ml, the 96% accuracy threshold, so were adjusted to 5 ng/ml for further statistical analysis. The number of such samples in each group is indicated by asterisks: *[1] = 10/20, *[2] = 3/20, *[3] = 15/20, *[4] = 1/20. SD: standard deviation. Metabolite abundance is reported in untreated samples (plasma or non-liquefied sputum).

Supplemental Table 5. Strains and plasmids used in this study

Name	Characteristics	Reference
***Mycobacterium tuberculosis* bacterial strains**		
H37Rv	Reference strain	Laboratory stocks
Δ*hsd*	Rv1106c::pKM464 derivative of H37Rv	This work
Δ*choD*	Rv3409c::pKM464 derivative of H37Rv	This work
H37Rv *att*::pCH89	H37Rv carrying the plasmid for chromosome complementation of the full *hsd* operon, as well as pKM461	This work
Δ*hsd* *att*::pCH89	Δ*hsd* carrying the plasmid for chromosome complementation of the full *hsd* operon, as well as pKM464	This work
**Plasmids**		
pKM461	P_{Tet}-Che9c RecT-Bxb1 Int; SacRB; TetR, colE1, oriM; Kan^R	Addgene ID = 108320; (2)
pKM464	Bxb1 *attB* site; *catP*; Hyg^R; colE1;	Addgene ID = 108322; (2)
pKP617	L5 integrating vector; Zeo^R	(8)
pCH89	pKP617-ZeoR-$Operon_{Rv1106c\text{-}Rv1109c}$ (integrating vector)	This work

Supplemental Table 6. Primers used in this study

<table>
<tr><th>Name</th><th>5’ Sequence</th><th>3’ Sequence</th><th>Use</th></tr>
<tr><td colspan="4">Mutant construction</td></tr>
<tr><td>HC291</td><td colspan="2">caccgcctcacccggcgcagtccggtgaccatatccaacgaccgcacgcttcctgatgcc
GGTTTGTCTGGTCAACCACCGCGGTCTCAGTGGTGTACGGTACAAACC
cgctgcagtatgacccccttta catcgggccagttaatcagtctctcaggtggcgtcagc</td><td>ORBIT deletion of Rv3409c</td></tr>
<tr><td>HC292</td><td colspan="2">cgccagcgggtgtccgatgtgctggccggcgacgaggcccaaaacggctaaggcaaggtt
GGTTTGTCTGGTCAACCACCGCGGTCTCAGTGGTGTACGGTACAAACC
ccagaattatctgaaactcaccacttgctgccccaggtcgctcggatgtgtgcgtcgacg</td><td>ORBIT deletion of Rv1106c</td></tr>
<tr><td>HC336</td><td></td><td>CGTCGGACGACCCGTTCGG</td><td rowspan="2">Screening ORBIT mediated deletion of Rv3409c</td></tr>
<tr><td>HC342</td><td></td><td>ccaccctacgttctctcgacg</td></tr>
<tr><td>HC338</td><td></td><td>CTGCCGGGACGAGCTGATGG</td><td rowspan="2">Screening ORBIT mediated deletion of Rv1106c</td></tr>
<tr><td>HC343</td><td></td><td>GGGTACGGACGCGGTGGATC</td></tr>
<tr><td colspan="4">Complementation plasmid</td></tr>
<tr><td>HC352</td><td>tatgcatcaattgatttatc</td><td>GCAGTAGCCACGCGGCTC</td><td rowspan="4">Cloning the Rv1106c region in pKP617</td></tr>
<tr><td>HC353</td><td>ggagtggtcggataacttac</td><td>CTACGGCTTGACTGTGGCG</td></tr>
<tr><td>HC354</td><td></td><td>gtaagttatccgaccactc</td></tr>
<tr><td>HC355</td><td></td><td>gataaatcaattgatgcatattaattaaatc</td></tr>
<tr><td>HC366</td><td></td><td>GTCGTTCACGGCTCTAGCTGATC</td><td rowspan="5">Sequencing the Rv1106c genetic region cloned</td></tr>
<tr><td>HC367</td><td></td><td>AGCCGACAGTTCCGACGCCT</td></tr>
<tr><td>HC368</td><td></td><td>GGACCTGCTGCCGTTTTCTG</td></tr>
<tr><td>HC369</td><td></td><td>ggagcagggcggattggacc</td></tr>
<tr><td>HC370</td><td></td><td>gggaaacgcctggtatcttt</td></tr>
</table>
